# Multiscale 3D Genome Rewiring during Mouse Neural Development

**DOI:** 10.1016/j.cell.2017.09.043

**Published:** 2017-10-19

**Authors:** Boyan Bonev, Netta Mendelson Cohen, Quentin Szabo, Lauriane Fritsch, Giorgio L. Papadopoulos, Yaniv Lubling, Xiaole Xu, Xiaodan Lv, Jean-Philippe Hugnot, Amos Tanay, Giacomo Cavalli

**Affiliations:** 1 Institute of Human Genetics, UMR 9002 of the CNRS and the Université de Montpellier, 34396 Montpellier, France; 2 Weizmann Institute of Science, Rehovot 76100, Israel; 3 BGI, Shenzhen, 518083 Shenzhen, China; 4 INSERM U1051, Institute for Neurosciences, Hôpital Saint Eloi, Université de Montpellier 2, 34090 Montpellier, France

**Keywords:** 3D genome architecture, Hi-C, neural differentiation, cortical development, transcription factors, Polycomb, enhancers, transcription

## Abstract

Chromosome conformation capture technologies have revealed important insights into genome folding. Yet, how spatial genome architecture is related to gene expression and cell fate remains unclear. We comprehensively mapped 3D chromatin organization during mouse neural differentiation *in vitro* and *in vivo*, generating the highest-resolution Hi-C maps available to date. We found that transcription is correlated with chromatin insulation and long-range interactions, but dCas9-mediated activation is insufficient for creating TAD boundaries *de novo*. Additionally, we discovered long-range contacts between gene bodies of exon-rich, active genes in all cell types. During neural differentiation, contacts between active TADs become less pronounced while inactive TADs interact more strongly. An extensive Polycomb network in stem cells is disrupted, while dynamic interactions between neural transcription factors appear *in vivo*. Finally, cell type-specific enhancer-promoter contacts are established concomitant to gene expression. This work shows that multiple factors influence the dynamics of chromatin interactions in development.

## Highlights

• Ultra-deep Hi-C during mouse neural differentiation, both *in vitro* and *in vivo*
• Transcription is correlated with, but not sufficient for, local chromatin insulation
• Polycomb network is disrupted, while novel contacts between neural TF sites appear
• Dynamic contacts among exon-rich gene bodies, enhancer-promoters, and TF sites

## Graphical Abstract

**Figure:**
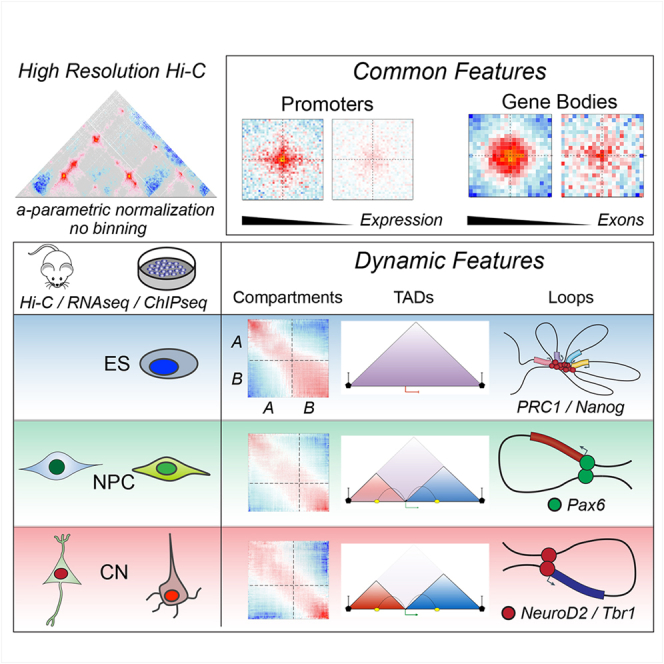

## Teaser

An ultrahigh resolution Hi-C map of mouse neural differentiation yields insights into the multiple factors that influence the dynamics of chromatin interactions during development.

## Introduction

Genome-wide 3D chromosome organization mapping technologies have revealed important insights on genome folding, but many fundamental questions remain open (Bonev and Cavalli, 2016, Dekker and Mirny, 2016, Dixon et al., 2016). Regulatory information, encoded in the spatial proximity of functional elements, exists at all levels—from histone-DNA interactions to interchromosomal and chromatin-lamina interactions. However, our understanding of the interaction dynamics at the smallest scales during developmental processes is currently limited.

At intermediate scales (100 kb to 1 Mb), a major discovery is the existence of topologically associating domains (TADs) (Dixon et al., 2012, Nora et al., 2012, Sexton et al., 2012). Domain insulation has been shown to be important for gene expression and physiology (Flavahan et al., 2016, Lupiáñez et al., 2015). It is known that architectural proteins such as CTCF and cohesin play a role (Haarhuis et al., 2017, Nora et al., 2017, Phillips-Cremins et al., 2013), and recently, novel models such as “loop extrusion” have been proposed to explain domain formation (Fudenberg et al., 2016, Sanborn et al., 2015). However, the relation between such models and transcription remains unclear.

At larger scales, interactions between domains are thought to give rise to two main types of spatial compartments referred to as “A” and “B” (Lieberman-Aiden et al., 2009), which are functionally related to gene expression and can switch extensively during differentiation (Dixon et al., 2015). The deposition of chromatin marks is thought to contribute to this process (Dixon et al., 2015, Nora et al., 2012), and recent evidence indicates that domains and compartment formation can be at least partially uncoupled and may result from different biological phenomena (Nora et al., 2017).

Hi-C is uniquely poised to probe all of these aspects of 3D chromatin architecture. However, given the enormous number of possible contacts in the genome, very few studies using mostly *in vitro* cultured cells have achieved high resolution (Rao et al., 2014). Furthermore, the major effect of the cell cycle on chromosome architecture (Nagano et al., 2017, Naumova et al., 2013) is rarely accounted for in 3D genome mapping studies. These issues have led to a conundrum, since depending on the method used, different studies highlight different and sometimes contrasting views of chromosome folding.

Here, we comprehensively mapped 3D chromatin organization at very high resolution using a well-defined cell differentiation system (Gaspard et al., 2008) in which we controlled for cell type heterogeneity and cell-cycle variation. We focused on neural differentiation and cortical development as a paradigm and performed ultra-deep coverage Hi-C on mouse embryonic stem cells (ESs), neural progenitors (NPCs), and cortical neurons (CNs). Furthermore, using transgenic mouse lines, we purified NPCs and CNs directly from the developing mouse embryonic neocortex *in vivo*. In total, we sequenced over 40 billion paired-end reads that resulted in more than 17 billion uniquely mapped contacts. Together with linear chromatin marks and RNA sequencing (RNA-seq), this rich dataset has allowed us to explore the relationship between gene expression, the epigenome, and 3D genome conformation. Our data demonstrate that cellular differentiation involves changes at all scales of folding, and in addition to CTCF-dependent processes, it includes major changes in Polycomb and in transcription factor (TF) interaction networks. This rich dataset provides a framework to study dynamic chromatin interactions and their influence on gene expression and cell fate.

## Results

### Global Reorganization of the 3D Genome during Neural Differentiation

**Figure S1:**
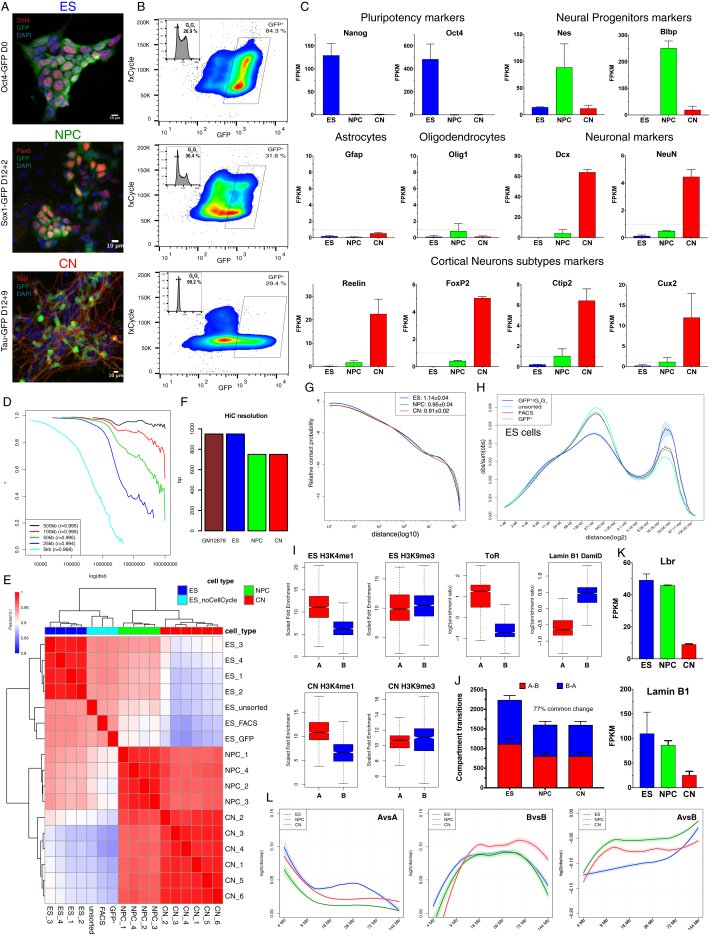
Ultra-Deep Hi-C during Mouse Neural Differentiation, Related to Figure 1 (A) Representative immunofluorescence images for the three cell lines used during this study. Neural differentiation is performed as described (Gaspard et al., 2008) and NPC and CN cells were obtained 12+2 or 12+9 days after changing to DDM media respectively. (B) FACS purification to select GFP^+^ population, which are also in the G_0_G_1_^+^ phase of the cell cycle based on DNA content. (C) Expression pattern of several markers for pluripotency, neural progenitors, differentiation or cortical neuronal subtypes. Data are represented as the mean ± SD based on two biological replicate RNaseq experiments. (D) Pearson’s correlation between two biological HiC replicates (ES1 and ES3), as a function of the genomic distance between interacting regions. (E) Pairwise Pearson’s correlation between Hi-C samples generated using the *in vitro* neuronal differentiation system (at 50Kb resolution and considering only contacts separated by at least 100Kb and not more than 2.6Mb). Note that the major separation occurs between cell types and also that ESs which were not sorted based on cell cycle phase (“ES_noCellCycle”) cluster separately. (F) HiC resolution achieved in this study, calculated exactly as described (Rao et al., 2014). The highest resolution Hi-C available so far – in human GM12878 cells (Rao et al., 2014) is shown as comparison. (G) Log-log contact probability as a function of the genomic distance. The exponent γ represents the mean slope ± SD of the best-fit line between 100Kb and 2Mb, multiplied by −1. (H) Contact probability as a function of the genomic distance in logarithmic bins (without dividing by bin size). Lines represent the mean values from biological replicates where available; semi-transparent ribbons show SEM. Note that while sorting itself does not have a major consequences on the contact distribution profile, samples with more cells in G_2_/M are characterized by a higher proportion of close-range *cis* contacts. (I) Enrichment for either ChIP-seq signal or replication timing (Hiratani et al., 2010)/ Lamin B1 DamID (Peric-Hupkes et al., 2010) where available, in the two compartments. (J) Number of compartment transitions as determined using the cis-Eigenvector 1 calculated at 100Kb resolution. Shown is also the ratio of common compartment borders that are also changed between ESs and CNs compared to ESs to NPCs (±100kb). (K) Expression of the Lamin B receptor (Lbr) and Lamin B1 during neural differentiation. (L) Contact enrichment represented as the log ratio between observed and expected contacts overlapping with the indicated domain type as a function of the genomic distances. Data were smoothed using loess regression. Lines represent the mean values from biological replicates; semi-transparent ribbons show SEM.

We used transgenic cell lines expressing GFP either from the Oct4, Sox1 (Ying et al., 2003), or Tau (*Mapt*) (Bibel et al., 2004) loci to obtain highly pure populations corresponding to the respective cell type: ES, NPC, or CN (Figures S1A–S1C). In addition, we employed a fluorescence-activated cell sorting (FACS) approach, which allowed us to purify G_0_G_1_ cells based on DNA content (Figures S1B and S1H).

**Figure 1:**
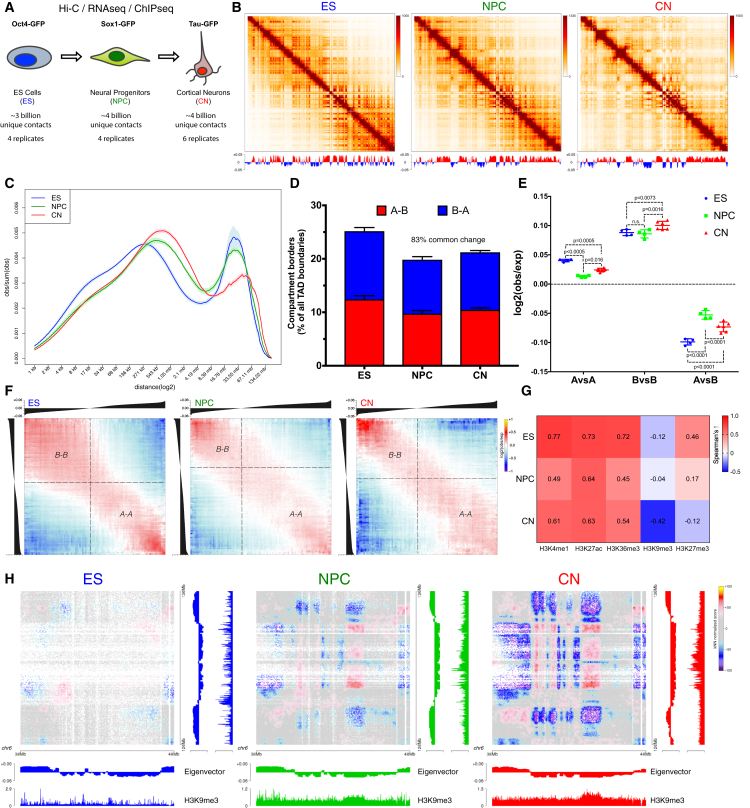
Global Reorganization of 3D Genome Architecture during Neural Development (A) Schematic representation of the *in vitro* system. (B) Observed contact matrices for chr3 at 250-kb resolution and the first eigenvector at 100-kb resolution. Scale bar is adjusted to account for the total coverage on chr3 in each cell type. (C) Contact probability in logarithmic bins. Lines: mean values from biological replicates; semi-transparent ribbons: SEM. (D) Number of borders between adjacent TADs of different type normalized by the total number of TAD boundaries. Error bars represent SD. Shown also is the percentage of common compartment borders that are also changed between ESs and CNs compared to between ESs and NPCs (± 100 kb). (E) Contact enrichment between domains from the same (“A” versus “A” or “B” versus “B”) and different (“A” versus “B”) type. Data are represented as a scatter dot plot showing the mean ± SD. Statistical significance is calculated using two-way ANOVA with Tukey’s test. (F) Average contact enrichment between pairs of 100-kb loci arranged by their eigenvalue (shown on top). (G) Spearman’s correlation between the eigenvector value and ChIP-seq signal enrichment in 100-kb bins. (H) Hi-C contact map between two B-type regions. Each point represents a contact, color-coded according to the density of the observed contacts around it, normalized by the density of the expected contacts (STAR Methods). See also Figure S1 and S2 and Tables S1 and S2.

**Figure S2:**
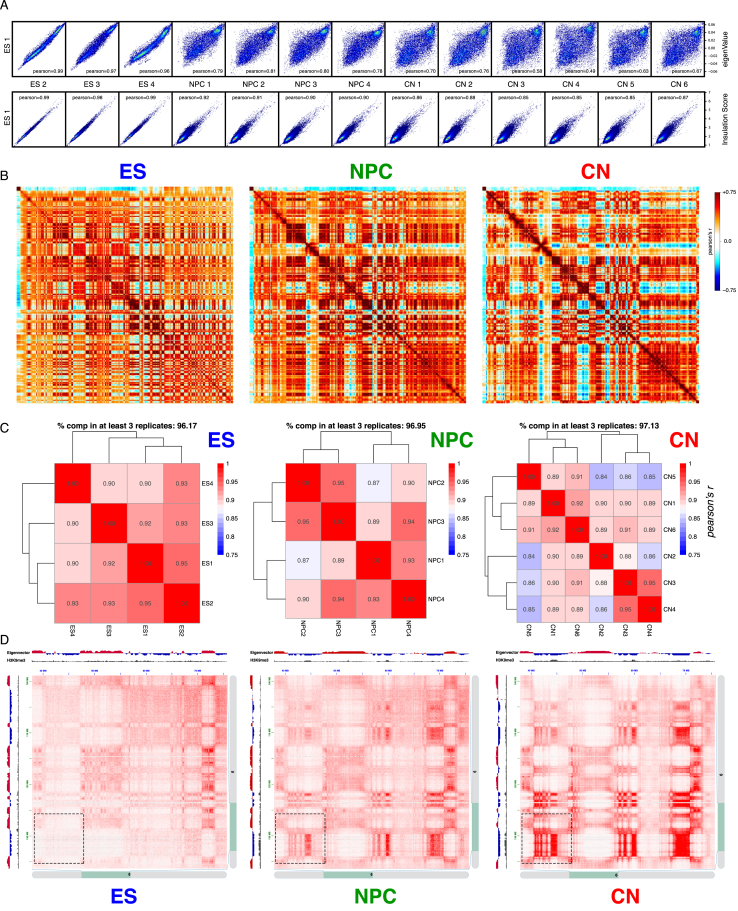
Hi-C Compartments and Reproducibility across Replicates, Related to Figure 1 (A) Example scatterplots showing the correlations between eigenvalues or insulation score between ES1 and the rest of the samples. (B) Pearson correlation Hi-C matrices for chr3 based on pooled data at 100kb resolution. (C) Classification of domains into A and B type is robust across replicates. Shown is the correlation when domains are assigned to the A- or the B- compartment in individual replicates and percentage of domains with identical classification in at least 3 replicates. (D) Hi-C contact map at 50kb resolution generated using Juicebox (Rao et al., 2014) showing increased interactions within the B-compartment during differentiation. Shown is also the eigenvector and the H3K9me3 ChIP-seq from the corresponding cell type. Region shown in Figure 1H is highlighted with a dashed square.

By using *in situ* Hi-C (Rao et al., 2014), we produced over 11 billion uniquely aligned contacts (Figure 1A and Table S1). Biological replicates were highly correlated at all resolutions (Figure S1D) and for each different cell type (Figures S1E and S2A). We reached a maximum resolution of 750 bp—the highest to date (Figure S1F).

Analysis of these Hi-C data showed a global reorganization in chromatin interactions during differentiation (Figures 1B, 1C, S1G, and S2B). We then identified TADs and classified them into two compartments: active (A) and inactive (B) (Figures S1I and S2C, Table S2, and STAR Methods). We observed that the number of compartment borders decreases with differentiation and confirmed these results using an analysis based on the eigenvector value (Lieberman-Aiden et al., 2009) (Figures 1D and S1J). Surprisingly, the interaction strength between A-type domains decreased, whereas contacts within the B compartment became stronger between NPCs and CNs (Figures 1E and S1L). Average contact enrichment between pairs of 100-kb loci arranged by their eigenvalue confirmed these results (Figure 1F). In addition, the correlation between signal enrichment and eigenvector value for active histone marks decreased during differentiation, while the anti-correlation between H3K9me3 and the eigenvector value became stronger (Figure 1G). Analysis of the contact map (STAR Methods) between several B-type regions also confirmed increased interaction strength during differentiation (Figures 1H and S2D).

In conclusion, a major genome-folding reprogramming occurs during neural differentiation with a progressive increase in compartment size, decreased interactions within the A compartment, and a concomitant increase in interactions between B-type domains.

### Transcription Is Correlated with but Not Sufficient to Induce Insulation at TAD Boundaries *De Novo*

**Figure S3:**
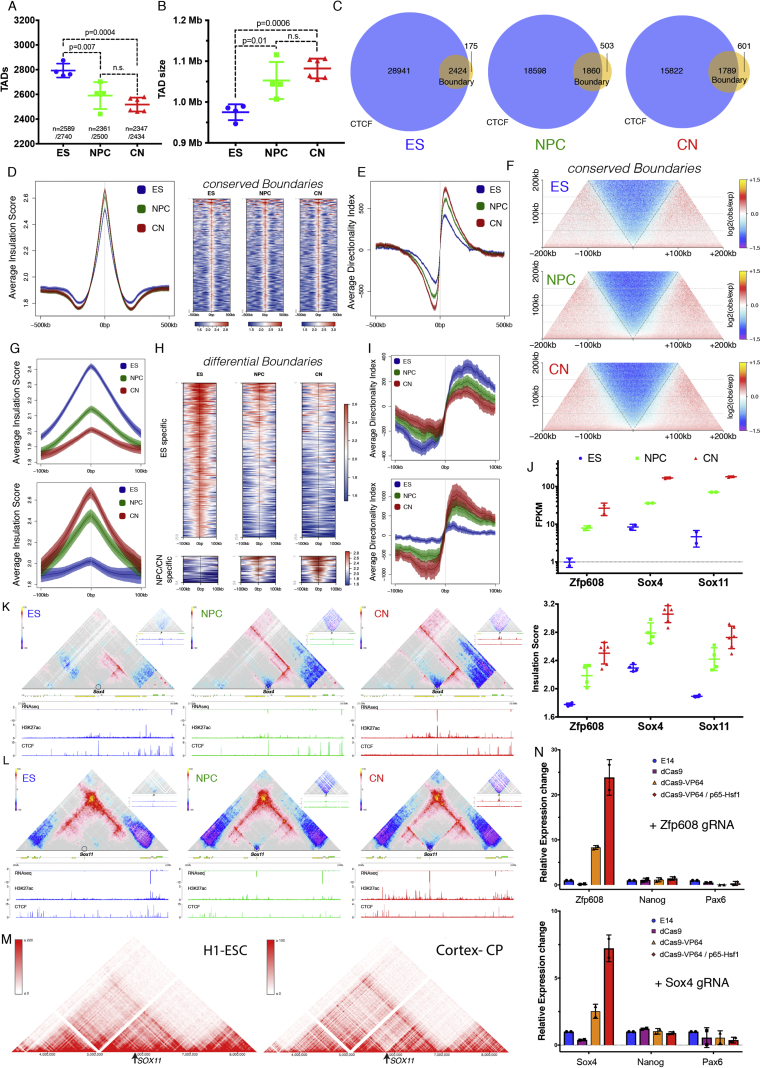
Cell-Type Specific TAD Boundaries Can Be CTCF-Independent and Are Frequently Correlated with Active Promoters, Related to Figure 2 (A) The number of TADs identified in this study. Data are represented as a scatter dot plot showing the mean ± SD. Shown is also the number of TAD boundaries identified in at least 3 biological replicates. (B) Average TAD size in the three cell types. Data are represented as a scatter dot plot showing the mean ± SD. (C) Overlap between TAD boundaries and CTCF sites in a 20Kb window. Multiple sites (boundaries or CTCF binding sites) within this window were counted only once. Note the gradual increase of CTCF^-^ TAD boundaries during differentiation. (D) Average insulation score and heatmaps in a 1Mb region around conserved TAD boundaries. Lines show mean values, while dark and light shaded ribbons represent SD and 95%CI respectively. (E) Average directionality index (DI) in a 1Mb region centered on conserved TAD boundaries. Lines show mean values, while dark and light shaded ribbons represent SD and 95%CI respectively. Note the gradual increase in insulation with differentiation. (F) Aggregate HiC maps centered on the conserved TAD boundaries. Data are presented as the log ratio of observed and expected contacts in 500bp bins. (G) Average insulation score a 200Kb region centered on either ES, or NPC/CN specific TAD boundaries. Lines show mean values, while dark and light shaded ribbons represent SD and 95%CI respectively. (H) Heatmaps representing the insulation score in a 200Kb region centered on either ES, or NPC/CN specific TAD boundaries. (I) Average DI a 200Kb region centered on either ES, or NPC/CN specific TAD boundaries. Lines show mean values, while dark and light shaded ribbons represent SD and 95%CI respectively. (J) Expression of *Zfp608, Sox4* and *Sox11* represented as the mean ± SD on a log10 scale. Also shown is the maximum insulation score at the Tss ± 10kb. (K) Hi-C maps in ∼3Mb region around *Sox4* gene (shaded). Shown are also RNaseq, H3K27ac and CTCF ChIP0seq tracks in each cell type. Scale bars represent RPM. Insets show a magnified view of the region around *Sox4* promoter (arrow). (L) HiC maps in ∼3Mb region around *Sox11* gene (shaded). Shown are also transcriptional output (RNaseq), H3K27ac and CTCF ChIP-seq tracks in each cell type. Scale bars represent RPM. Insets show a magnified view of the region around *Sox11* promoter. (M) Hi-C maps around the human *SOX11* locus obtained through the *3D Genome Browser* at http://www.3dgenome.org/ZMcSZ/ using publically available data (Dixon et al., 2015, Won et al., 2016). (N) qPCR showing the relative expression of the targeted gene locus upon CRISPR-dCas9 activation. E14 represents ESs infected only with the guideRNA, dCas9/dCas9-VP64/dCas9-VP64+p65-Hsf1 are stable cell lines expressing the indicated form of dCas9, infected with the indicated guide RNA. The values of each replicate (N = 2) is shown together with the mean ± SD. The expression of ES (Nanog) or NPC (Pax6) marker gene is shown as a comparison.

We then examined TADs and found that their number decreased during differentiation (Figure S3A), with a corresponding increase in size (Figure S3B). Analysis of TAD boundaries revealed that the proportion of the CTCF-less borders increased during differentiation (Figure S3C). In addition to CTCF, cohesin and transcription have also been suggested to play a role in boundary formation (Dixon et al., 2012, Fudenberg et al., 2016), although given low resolution of TAD boundaries in previous studies, it has been difficult to disentangle the role of these other factors from that of CTCF.

**Figure 2:**
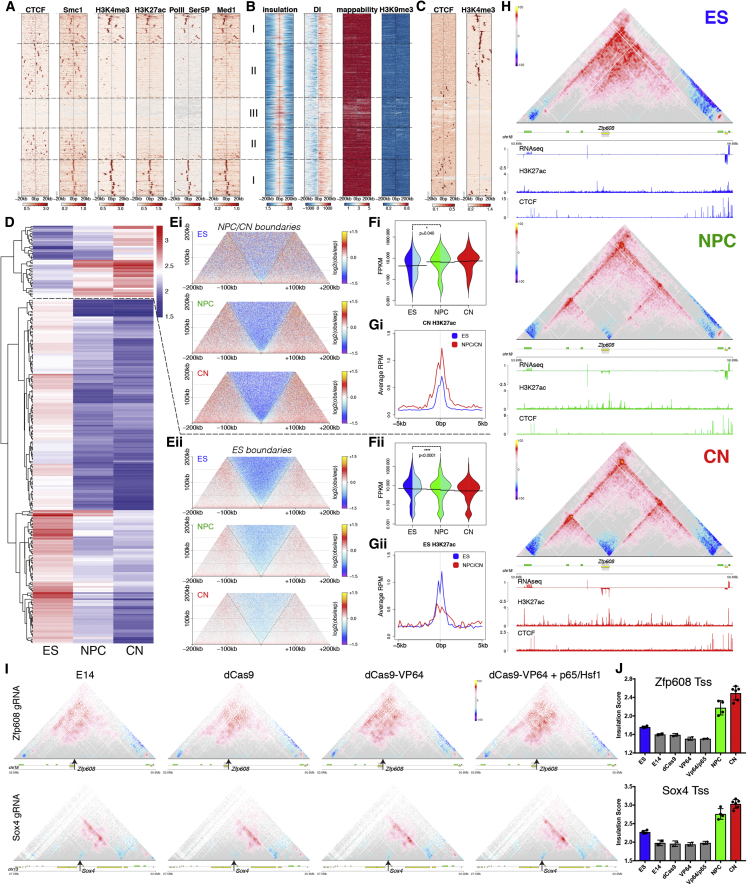
Transcription Is Correlated with but Not Sufficient to Cause Insulation at TAD Boundaries (A–C) Signal enrichment centered on TAD boundaries in ES (A and B) or CN (C) cells. Rows were ordered using hierarchical clustering. The three main classes (Tss: I, CTCF: II and repeats: III) are highlighted. (D) Hierarchical clustering of differential TAD boundaries based on the insulation score. We denote the two major clusters as ES-specific boundaries: n = 259, and neural-specific boundaries: n = 54. (E) Aggregate Hi-C maps centered on either neural-specific (Ei) or ES-specific (Eii) TAD boundaries. (F) Beanplots showing expression of genes in close proximity (< 10 kb) of a differential boundary. Each half of the bean represents a separate RNA-seq replicate. Lines show the mean value per replicate. p value is calculated using Wilcoxon rank-sum test. (G) Average enrichment of H3K27ac in CNs (Gi) or ESs (Gii) at differential TAD boundaries. (H) Example of a novel neural boundary at the *Zfp608* locus. (I) Hi-C maps at the *Zfp608* and *Sox4* loci upon CRISPR-dCas9 gene activation. (J) Average insulation score at the Zfp608 or Sox4 Tss ± 5 kb. Data are presented as mean ± SD. The values based on the high-resolution Hi-C samples (ES, NPC, and CN) are shown as comparison. In all panels, genes transcribed in the forward direction are represented by green rectangles and genes in the reverse orientation are represented by yellow. See also Figure S3 and Table S2.

We thus examined the signal enrichment of CTCF, of Smc1, and of several transcription-related chromatin marks around TAD borders in ESs. We observed three classes of boundaries: CTCF-positive boundaries, CTCF-negative boundaries close to active gene promoters, and a set of boundaries without enrichment of active marks (Figure 2A). Importantly, cohesin was found both at CTCF-positive boundaries, where it colocalized with CTCF, and on CTCF-negative borders marked by active transcription (Figure 2A). The third set of boundaries appear to represent repeats (Figure 2B), consistent with previous findings (Dixon et al., 2012). The existence of three types of boundaries was confirmed in CN cells (Figure 2C).

TAD boundaries were reported to be mostly conserved between different cell types and even across species (Dixon et al., 2012, Rao et al., 2014), yet the mechanisms contributing to developmentally regulated borders remain poorly understood. Highly conserved boundaries in our system were characterized by a small but consistent increase in insulation during differentiation (Figures S3D–S3F). We classified developmentally regulated TAD boundaries into ES-specific and neural-specific regions (Figure 2D) and verified that these borders represent a genuine loss or gain of insulation, respectively, using aggregate Hi-C maps (Figure 2E), insulation score (Figures S3G and S3H), and directionality index (DI) (Figure S3I). We found that genes located in close proximity to the neural-specific TAD boundaries tend to be upregulated (Figures 2Fi and 2Gi), while genes located close to ES-specific borders are less active in differentiated cells (Figures 2Fii and 2Gii).

We examined one such neural-specific region, where a novel TAD boundary was formed at the transcriptional start site (Tss) of *Zfp608* during differentiation (Figure 2H), concomitant with the activation of the gene (Figure S3J) but in the absence of CTCF binding. We also examined two other neural genes: *Sox4* and *Sox11*. CTCF was bound to the *Sox4* promoter already in ESs, yet insulation dramatically increased only when the gene became expressed at the NPC stage (Figures S3K and S3J). For *Sox11*, we also observed increased CTCF binding at its promoter (Figure S3L). Remarkably, the changes in insulation were highly similar to the changes in transcription across all three genes (Figure S3J). Next, we asked whether some aspects of novel TAD boundary formation are also conserved in human cortical development. Despite the much lower resolution of the Hi-C maps available (Dixon et al., 2015, Won et al., 2016), we observed a similar increase in insulation around *SOX11* (Figure S3M).

Finally, we asked if RNA polymerase II (Pol II) recruitment is sufficient to cause chromatin insulation. We used the CRISPR-dCas9 system (Konermann et al., 2015) to activate gene expression at the endogenous *Zfp608* and *Sox4* loci, which did not cause precocious neural differentiation (Figure S3N). We then performed Hi-C and found that despite more than 10- to 20-fold gene activation, which is comparable to expression levels in NPCs, there was no significant change in the contact maps or the insulation score at the examined loci (Figures 2I and 2J). Therefore, transcriptional activation per se appears insufficient to cause chromatin insulation and create a TAD boundary.

In summary, transcription is correlated to chromatin insulation in a manner that can be independent from CTCF binding. The proportion of CTCF-less TAD boundaries, located close to or at active gene promoters, increases during neural differentiation. However, transcription is not sufficient to cause chromatin insulation at these loci.

### Dynamic CTCF-Based Loops and Insulation during Neural Differentiation

Chromatin insulation occurs at multiple levels and leads to a hierarchical 3D nuclear organization, whereby smaller, less insulated regions (called also sub-TADs [Flavahan et al., 2016, Phillips-Cremins et al., 2013] or contact domains [Rao et al., 2014]) are nested inside TADs, which are usually bigger and more strongly insulated (Dixon et al., 2012, Nora et al., 2012, Sexton et al., 2012).

**Figure 3:**
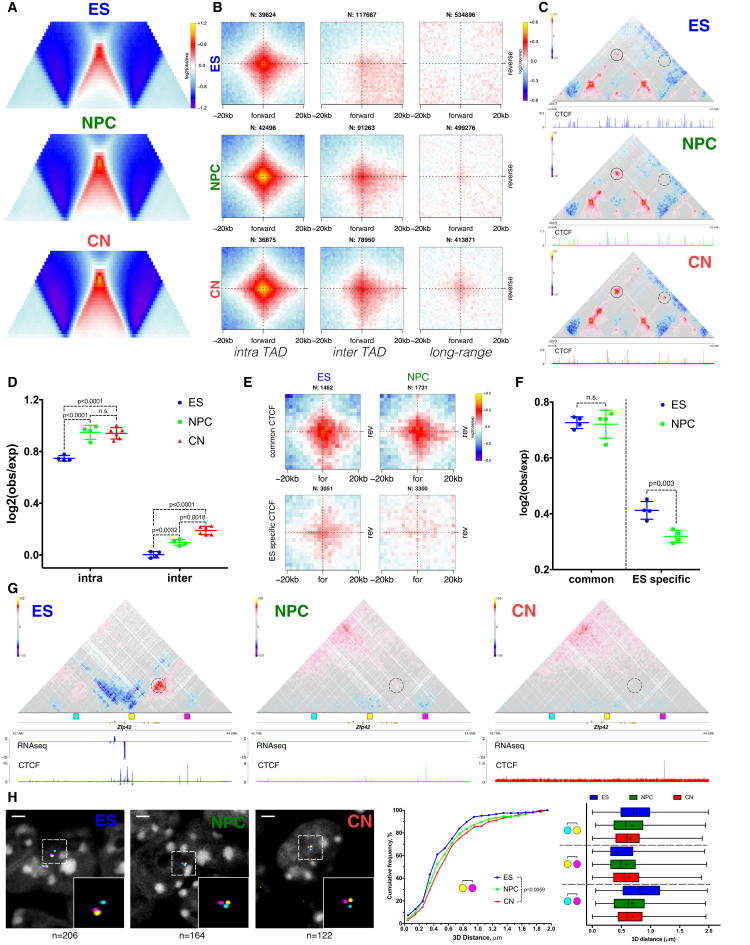
Stronger Loops between Convergent CTCF Sites and Dynamic Chromatin Contacts at Cell-Type Specific CTCF Sites (A) Average TAD representation in each cell type. Note the increase in contact enrichment at the domain loop. (B) Aggregate Hi-C map around pairs of convergent CTCF binding sites in each cell type. (C) Example of a Hi-C map at a conserved region. Note the increase of contact enrichment between CTCF sites (between domain boundaries: circle, inter-TAD: dashed circle). (D) Scatter dot plot showing the mean ± SD contact enrichment between convergent CTCF sites. Statistical significance is calculated using two-way ANOVA with Tukey’s test. (E and F) Aggregated contacts and quantification around intra-TAD pairs of convergent CTCF binding sites either present in both ESs and NPCs (“common CTCF”) or ES specific. Data are represented as a scatter dot plot showing the mean ± SD. Statistical significance is calculated using two-way ANOVA with Tukey’s test. (G) Hi-C contact maps at the *Zfp42* locus. Regions examined by FISH are indicated with colored squares. (H) Representative 3D-DNA FISH images (z-slice) and quantification for the regions shown in (G). In all panels, n refers to the number of pairs examined. See also Figure S4 and Table S4.

**Figure S4:**
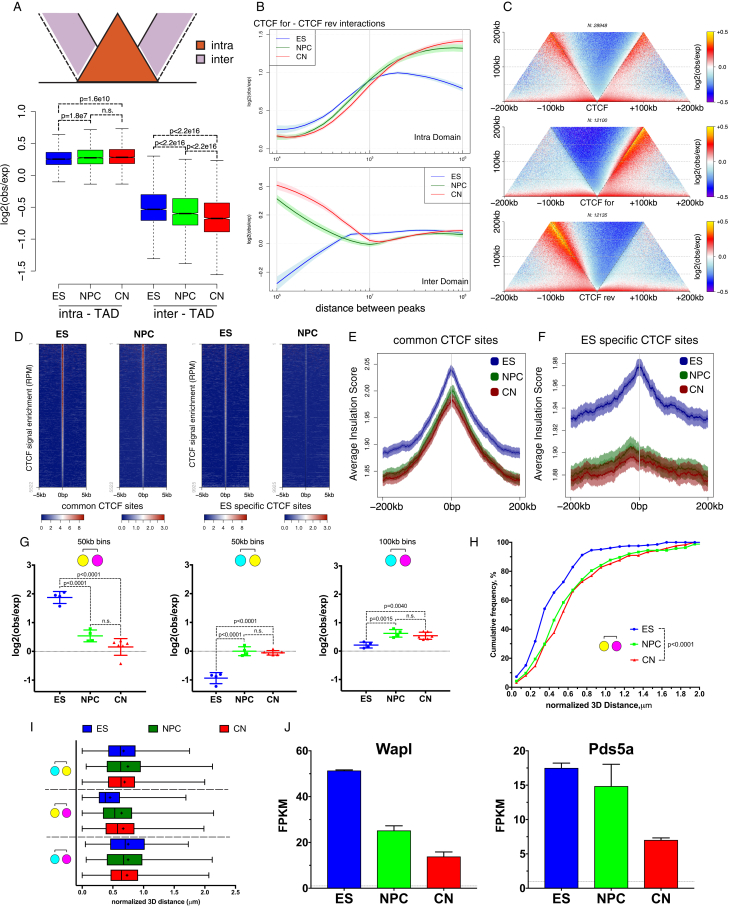
Dynamic CTCF Contacts Contribute to Cell-Type Specific 3D Genome Architecture, Related to Figure 3 (A) Contact enrichment intra- and inter-TAD during differentiation (STAR Methods). Data represented as a boxplot based on the intra- and inter- values per TAD. Statistics are calculated using the unpaired Wilcoxon test. (B) Contact enrichment between pairs of convergent CTCF sites represented as the log ratio between observed and expected contacts as a function of the genomic distances. Data were smoothed using loess regression. Lines represent the mean values from biological replicates; semi-transparent ribbons show SEM. (C) Aggregate Hi-C maps in ESs centered on the top 30000 CTCF binding sites based on ChIP enrichment in ESs (separated based on the orientation of the CTCF motif). (D) Heatmaps showing CTCF ChIP-seq signal enrichment around either common sites (present in ESs and NPCs), or ES-specific sites. (E and F) Average insulation score centered around either conserved or ES specific CTCF sites. Lines show mean values, while dark and light shaded ribbons represent SD and 95%CI respectively. (G) Quantification of the contact enrichment between the regions shown in Figure 3G between either 50kb or 100kb bins centered on the middle of the FISH probe region. Data are presented as a scatter dot plot showing the mean ± SD. Statistical significance is calculated using one-way ANOVA with Tukey’s correction. (H) Distribution of the normalized 3D FISH distances between the regions showed in Figure 3G. (STAR Methods). (I) Quantification of the normalized FISH distances between the regions showed in Figure 3G. (J) Expression of the cohesin unloading complexes Wapl and Pds5a decreases during neural differentiation. Shown is the mean ± SD based on the RNaseq data.

We observed that average intra-TAD contacts become stronger, while inter-TAD contacts appear to be depleted during differentiation (Figures 3A and S4A). In addition, the average contact enrichment at the loop between two TAD boundaries also increased (Figure 3A). As most of the TAD boundaries are bound by CTCF, we also quantified the interaction strength between convergent CTCF sites using aggregate Hi-C maps (STAR Methods). We observed strong enrichment as expected (Guo et al., 2015, Rao et al., 2014), and the loops were constrained primarily inside TADs and rarely crossed TAD boundaries (Figures 3B and 3D). However, the overall enrichment of CTCF-mediated contacts increased with neural differentiation, and even inter-domain contacts became apparent (Figures 3B–3D and S4B). In addition, CTCF sites insulated adjacent regions asymmetrically based on their motif orientation (Figure S4C).

As many as 40–50% of the CTCF sites may change in different cell types (Chen et al., 2012). To test whether differences in CTCF binding could be related to cell-type specific chromatin interactions, we compared CTCF binding between ESs and NPCs (Figure S4D). Conserved CTCF were characterized by strong interactions in the convergent orientation (Figures 3E and 3F) and by strong insulation both in ESs and NPCs (Figure S4E). In contrast, both the contact enrichment and the insulation between ES-specific CTCF regions were decreased in NPCs (Figures 3E, 3F and S4F).

Next, we examined one of the regions with predicted ES-specific CTCF interaction (Figure 3G, dashed circle). During differentiation, only the forward CTCF site became inaccessible to CTCF, and the chromatin loop disappeared (Figure 3G). To confirm this result, we used three-color fluorescence *in situ* hybridization (FISH) with probes at the forward CTCF site, reverse site, and an equidistant upstream region (Figure 3G). Consistent with Hi-C (Figure S4G), only the 3D FISH distance between the pair of probes containing the forward and the reverse CTCF site increased during differentiation, while the other probe pairs displayed the opposite effect (Figures 3H, S4H, and S4I).

### Active Gene Promoters and the Bodies of Exon-Rich Genes Interact at Multiple Genomic Scales

**Figure 4:**
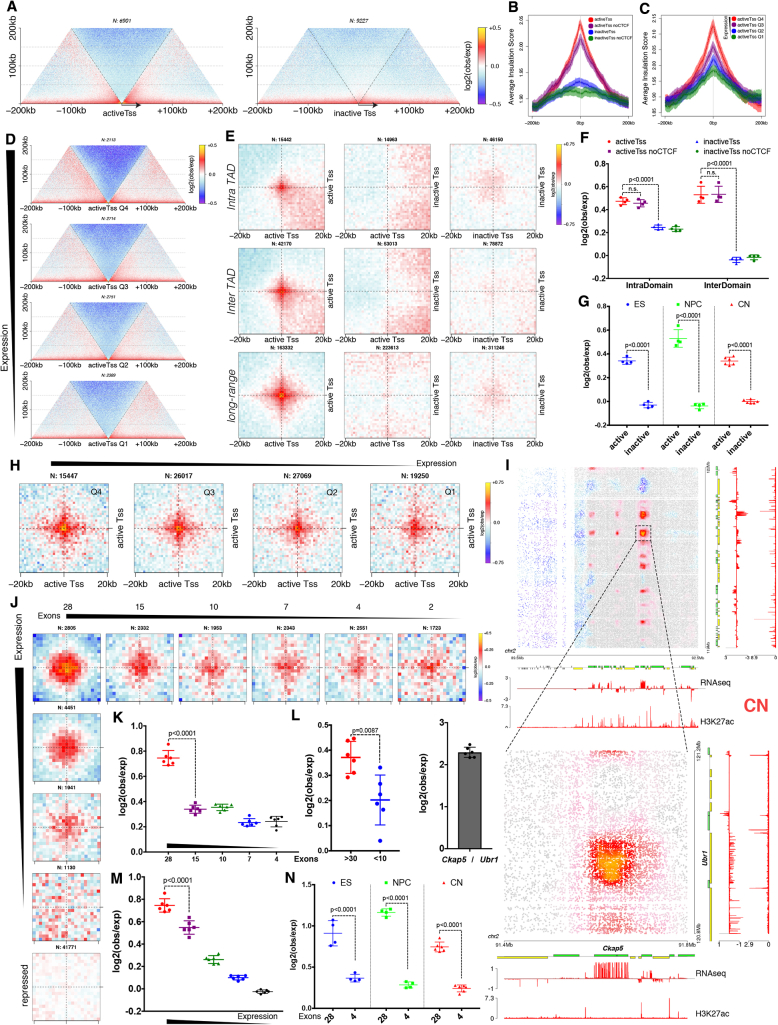
Long-Range Interactions between Active Promoters and Gene Bodies with Many Exons (A) Aggregate Hi-C maps in NPCs centered on either active or repressed gene promoters with no CTCF binding site within ± 5 kb. (B) Average insulation score centered on gene promoters in NPCs. Lines show mean values; dark and light shaded ribbons represent SD and 95% confidence interval, respectively. (C and D) Average insulation score (C) and aggregate Hi-C maps (D) centered on active gene promoters in NPCs separated into quartiles based on expression. (E) Aggregate Hi-C contact maps around pairs of gene promoters in NPCs with no CTCF binding site within ± 5 kb. (F and G) Quantification of the contact strength between pairs of either active or repressed Tss (F) or between inter-TAD pairs of either active or repressed Tss (G). Data are represented as a scatter dot plot showing the mean ± SD. Statistical significance is calculated using two-way ANOVA with Tukey’s test. (H) Aggregate Hi-C contact maps around pairs of active gene promoters in NPCs separated into quartiles based on expression. Shown are long-range (2- to 10-Mb) inter-TAD interactions. (I) Example of clusters of long-range loops between gene bodies in CNs. Also shown is a zoomed-in interaction between the *Ckap5* and *Ubr1* genes and quantification of the contact enrichment. Genes transcribed in the forward direction are represented by green rectangles and genes in the reverse orientation are represented by yellow. (J) Aggregate Hi-C maps between long-range (10- to 50-Mb) pairs of genes (central bin represents the middle of the gene) separated in quantiles based on either number of exons (shown is the average number of exons in a quantile) or gene expression. Shown are 160 × 160 kb windows with a bin size of 8 kb. (K–N) Quantification of the contact enrichment between genes (25-kb bins around the center of the gene) by the average number of exons (K), by the number of exons in genes with comparable length (L), by average expression (M), and in different cell types (N). Data are represented as a scatter dot plot showing the mean ± SD. Statistical significance is calculated using two-way ANOVA with Tukey’s test. In all panels, n refers to the number of intervals or pairs examined. See also Figure S5.

**Figure S5:**
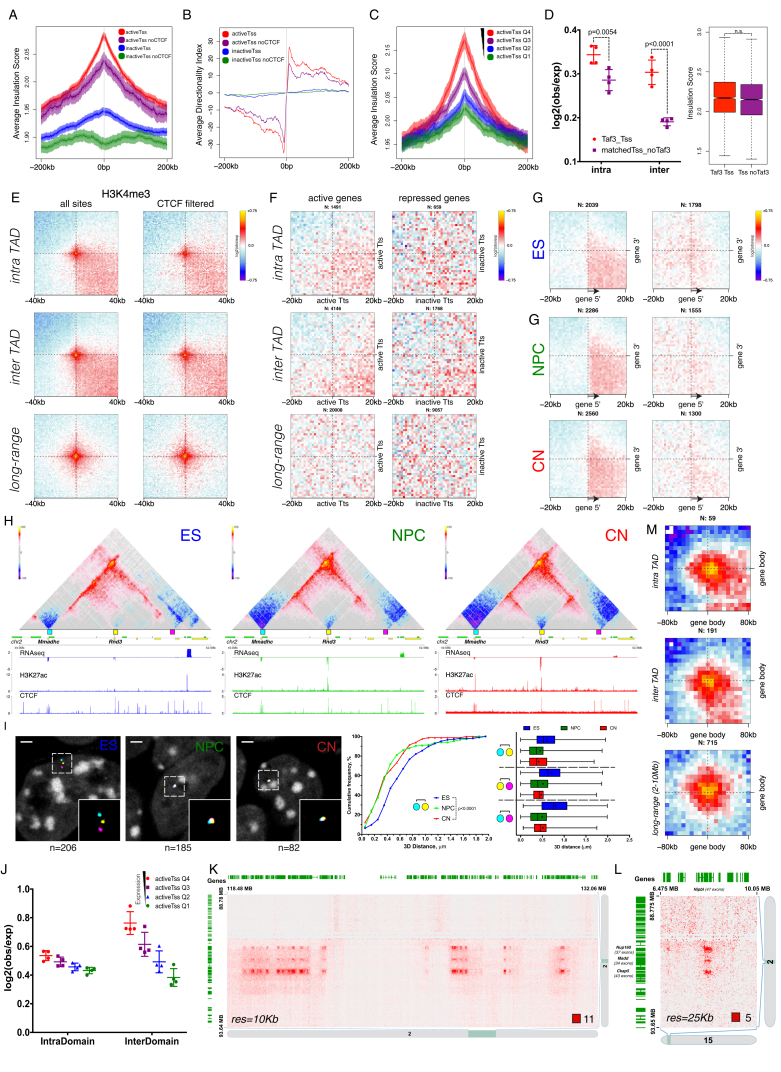
Transcription-Associated Contacts and Local Chromatin Insulation around Gene Promoters, Related to Figure 4 (A) Average insulation score centered on gene promoters in ESs. Lines show mean values, while dark and light shaded ribbons represent SD and 95%CI respectively. (B) Average DI centered on different types of gene promoters in NPCs. (C) Average insulation score centered on active gene promoters in ESs separated into quartiles based on expression values in ESs. (D) Quantification of the contact enrichment and insulation at Tss which are bound by Taf3 or expression matched Tss which are not bound Taf3 in ESs. Statistics are calculated using two-way Anova with Sidak’s correction (contacts) or the Wilcoxon’s unpaired test (insulation). (E) Aggregate Hi-C maps in NPCs between pairs of either all H3K4me3 sites or filtered for the presence of CTCF binding site within ± 5Kb. (F) Aggregate Hi-C contact maps around pairs of gene transcription termination sites separated into active or inactive based on expression. (G) Aggregate Hi-C maps showing the interactions between matched gene promoters and transcription termination sites. Only genes longer than 50Kb and not spanning a TAD border are examined. (H) Hi-C contact maps showing ∼2.5Mb region around the *Rnd3* gene. Regions examined by FISH are indicated with colored squares. (I) Representative 3D- DNA FISH images (z-slices) and quantifications showing the decrease in distance between the regions shown in (H). (J) Quantification of the contact enrichment between pairs of active gene promoters separated into quartiles based on expression. Data are represented as a scatter dot plot showing the mean ± SD. (K and L) Cluster of long-range chromatin loops either in *cis* (K) or in *trans* (L), visualized using Juicebox (Rao et al., 2014). (M) Aggregate Hi-C maps between pairs of genes in the top expression and number of exons quantile based on Figure 4J.

We wondered if gene expression, local insulation, and chromatin contacts are also correlated even outside TAD borders. Using aggregate Hi-C maps, we identified a strong preference for insulation centered on active Tss, but not on inactive ones, a phenomenon that was not dependent on CTCF binding (Figures 4A, 4B, S5A, and S5B).

Next, we asked whether differences in insulation are also correlated with expression levels. We found that insulation scaled gradually with gene expression based on the insulation score (Figures 4C and S5C) and aggregate Hi-C maps (Figure 4D). Interestingly, general TFs such as Taf3 (Liu et al., 2011) appear to enhance interaction between promoters but had little effect on insulation (Figure S5D).

Interactions between active promoters have been observed with other methods (chromatin interaction analysis by paired-end tag sequencing [ChIA-PET] and promoter-capture Hi-C) (Li et al., 2012, Schoenfelder et al., 2015a, Tang et al., 2015), but due to the selectivity of these approaches, it is unclear to what extent promoter interactions are enriched above the local genomic average. We observed that interactions occur exclusively between the promoters of active genes (Figures 4E and S5E) and that active chromatin loops span across domains and can be observed even at long distances (Figure 4E), in contrast with CTCF-mediated contacts. Furthermore, most of these interactions were not mediated by CTCF (Figure 4F) and were readily identified in all three cell types (Figure 4G). Importantly, we observed no central enrichment when we performed the same analysis using instead 3′ gene ends (Figure S5F), showing that these interactions were specific to promoters and were correlated with the level of expression (Figures 4H and S5J). As an example verifying these observations, we show the contact maps at the locus of the neuronal gene *Rnd3* (Figures S5H and S5I).

Previous studies suggested that active promoters might engage in loops with their cognate 3′ gene ends in a process that might facilitate recycling of RNA pol II after the end of transcription (Tan-Wong et al., 2012). Aggregate Hi-C maps of intragenic contacts showed that active promoters contact the whole transcribed region of the gene (Figure S5G), suggesting that the contacts with the promoter are maintained throughout the act of transcription, and not just at the time of transcription termination.

Surprisingly, we also identified clusters of very long-range (> 30 Mb) chromatin contacts between bodies of expressed genes in *cis* (Figures 4I and S5K) and also in *trans* (Figure S5L). Unexpectedly, the contact enrichment between gene bodies was highly correlated not only with expression levels, but also with the number of exons per gene (Figures 4J, 4K, and 4M), and was evident across a large range of genomic distances (Figure S5M) in all three cell types (Figure 4N). Importantly, this effect was stronger for genes with more splicing events, even when we controlled for gene length (Figure 4L).

In summary, promoters of active genes are characterized by strong insulation, which is correlated with transcriptional activity, but is mostly CTCF independent. Active Tss maintain contacts with the gene body and, contrary to CTCF interactions, can interact with other transcribed Tss across TAD boundaries. Finally, actively transcribed genes with a large number of exons engage in long-range chromatin loops in clusters both in *cis*- and in *trans*.

### Polycomb-Mediated Interactions Are Disrupted Independently of H3K27me3 during Neural Differentiation

**Figure 5:**
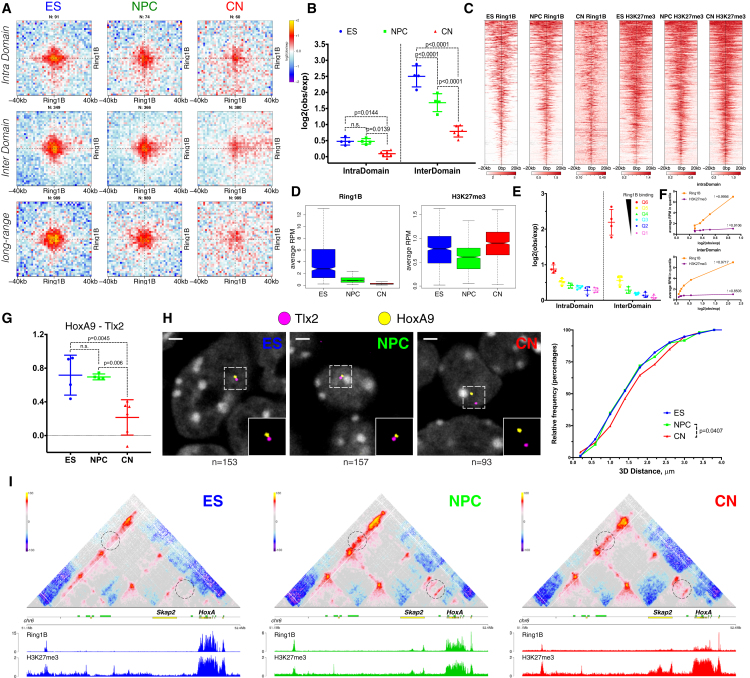
Contacts between Polycomb-Bound Regions Are Disrupted during Neural Differentiation (A and B) Aggregate Hi-C maps and quantification between pairs of regions bound by Ring1B in all three cell types. n refers to the number of pairs examined. Data are represented as a scatter dot plot showing the mean ± SD. Statistical significance is calculated using two-way ANOVA with Tukey’s test. (C and D) Heat maps and quantification showing the ChIP-seq signal enrichment centered on common Ring1B sites. Note the decrease in Ring1B binding, with no accompanying change in H3K27me3. (E) Quantification of the contact strength between pairs of the top 3,000 Ring1B binding sites in ESs divided into 6 quantiles based on Ring1B enrichment. (F) Plots of the average contact enrichment versus the average ChIP enrichment in ESs for either Ring1B or H3K27me3 in each quantile. Shown is also the Pearson correlation coefficient. (G) Quantification of the contact strength between a long-range Polycomb-associated contact (*Tlx2* and *HoxA9*) based on Hi-C data. (H) Representative 3D-DNA FISH images (z-slice) and quantification showing the increase in distance between *Tlx2* and *HoxA9* during differentiation. Statistics are calculated using the Kolmogoroc-Smirnov test. (I) Hi-C contact maps at the *HoxA* cluster. NPC-specific contact between *Skap2* and the *HoxA* cluster or an upstream Ring1B binding site are highlighted with a dashed circle. See also Figure S6 and Table S4.

**Figure S6:**
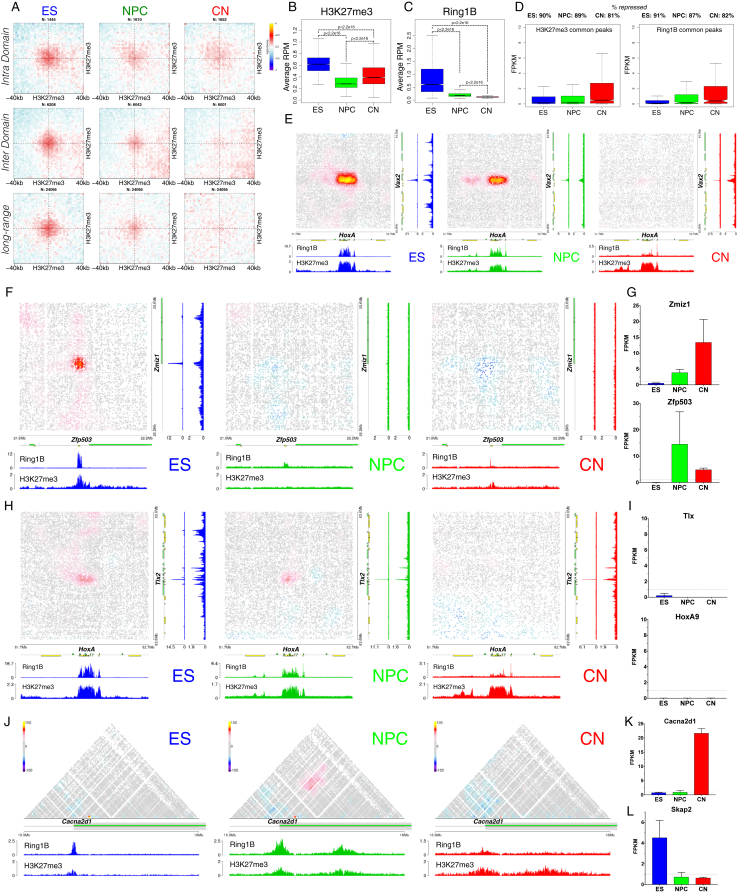
Dynamic Long-Range Contacts between Polycomb-Bound Regions Are Disrupted during Differentiation, Related to Figure 5 (A) Aggregate HiC maps between pairs of regions bound by H3K27me3 in all three cell types. (B and C) Quantification of the average enrichment of either Ring1B or H3K27me3 in each cell type on common H3K27me3 sites. Statistics are calculated using Wilcoxon’s unpaired test. (D) Expression (mean FPKM) of genes within 10Kb of either common H3K27me3, or Ring1B binding sites. The percentage of repressed or weakly expressed genes (FPKM ≤ 5) is also indicated. (E) HiC contact maps showing long-range interaction between the *HoxA* cluster and *Vax2*. (F) HiC contact maps showing long-range interaction between the *Zfp503* and *Zmiz1* promoter. (G) Expression of the *Zmiz1* and *Zfp503* genes, represented as the mean ± SD. (H) HiC contact maps showing long-range interaction between the *HoxA* cluster and *Tlx2*. Shown are also the Ring1B and H3K27me3 ChIP-seq tracks in each cell type. (I) Expression of the *Tlx2* and *Hoxa9* genes, represented as the mean FPKM value from two replicates. (J) HiC contact maps showing ∼150Kb region around *Cacna2d1* promoter. Note the appearance of NPC-specific Ring1B/H3K27me3 site inside *Cacna2d1* gene body, which coincides with an interaction between this region and the *Cacna2d1* promoter. (K and L) Expression of the *Cacna2d1* or *Skap2.*

Polycomb-mediated interactions have been previously reported in mouse ESs using a Capture-C approach (Schoenfelder et al., 2015b) and are a prominent feature in *Drosophila* (Bantignies et al., 2011). Unexpectedly, we observed a strong decrease in contact intensity between common H3K27me3 sites, which was especially notable at long distances (Figure S6A). This loss of contacts correlated better with Ring1B than with H3K27me3 on these sites (Figures S6B and S6C). In ESs, strong contact enrichment between conserved Ring1B sites, which decreased during differentiation, was evident in both intra- and inter-domain pairs and also across long distances (Figures 5A and 5B). Although the vast majority of these shared Ring1B sites were marked by both Ring1B and H3K27me3 (Figure 5C), the absolute levels of Ring1B decreased during differentiation (with only a mild effect on gene expression [Figure S6D]), while H3K27me3 levels did not (Figure 5D). Finally, the strength of pairwise contacts was highly correlated to the level of Ring1B rather than that of H3K27me3, even in ESs (Figures 5E and 5F). These findings are illustrated at the *HoxA*-*Vax2* (Figure S6E), *Zfp503*-*Zmiz1* (Figures S6F and S6G) and *Hoxa9-Tlx2* genomic loci (Figures 5G, 5H, S6H, and S6I).

As the majority of strong Polycomb-bound regions are on gene promoters, we examined whether their interaction might depend on paused Pol II that is located at bivalent gene promoters (Min et al., 2011). To address this question, we examined NPC-specific Ring1B sites. *Cacna2d1* is repressed in ESs and NPCs (Figure S6K). Importantly, a loop was formed specifically in NPCs between the *Cacna2d1* promoter and another NPC-specific Ring1B site located ∼500 kb downstream in the gene body (Figure S6J). *Skap2* is a gene expressed in ESs where, as expected, it did not contact the adjacent HoxA complex (Figure 5I). In NPCs, *Skap2* became repressed (Figure S6L), bound by Ring1B and H3K27me3, and acquired strong interactions with the HoxA cluster (Figure 5I). Based on these results, we concluded that Polycomb-mediated chromatin contacts are not exclusive to bivalent gene promoters and that they are unlikely to be a consequence of paused Pol II at bivalent Tss.

Collectively, these data suggest that Polycomb-mediated interactions are among the strongest in mouse ESs but become progressively disrupted with neuronal differentiation. However, Polycomb-mediated interactions are maintained or even enhanced at a small subset of genes in a manner that is highly correlated with the levels of Ring1B bound to its target loci.

### Cell-Type Specific Chromatin Folding Is Organized around the Neural Transcription Factors Pax6, NeuroD2, and Tbr1 *In Vivo*

**Figure S7:**
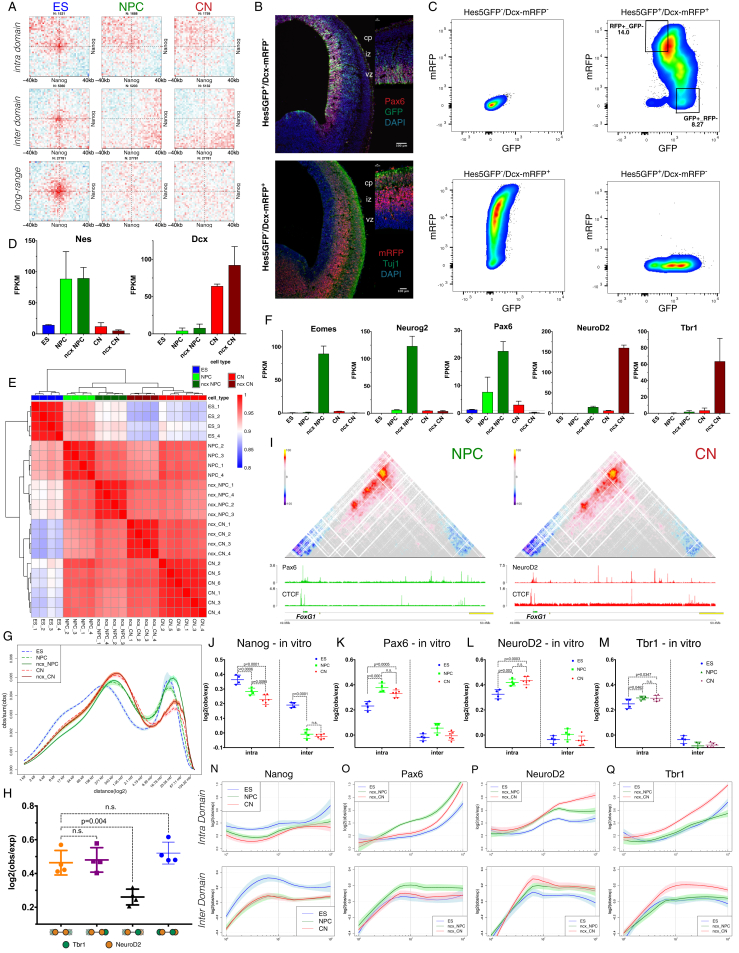
*In Vivo* Hi-C Reveals a Role for Neuronal Transcription Factors in Organizing Chromatin Interactions, Related to Figure 6 (A) Aggregate Hi-C maps between pairs of regions bound by Nanog in ESs. (B) Coronal section from E14.5 telencephalon (from Hes5GFP^+^/Dcx-mRFP^-^ or Hes5GFP^-^/Dcx-mRFP^+^ littermates) showing the overlap between the apical progenitor marker Pax6 and GFP, or between mRFP and the neuronal marker Tuj1. (C) FACS approach to purify GFP^+^/RFP^-^ (referred to as “ncx NPC” henceafter) and GFP^-^/RFP^+^ (“ncx CN”) populations from the same embryonic brains. (D) Expression of a neural progenitor (*Nes*) or neuronal marker (*Dcx)* either *in vivo* or *in vitro*. Data are represented as the mean ± SD from two biological replicates. (E) Pairwise Pearson’s correlation between Hi-C samples (at 50Kb resolution and considering only contacts separated by at least 100Kb and not more than 2.6Mb). Note that the major separation occurs between cell types. (F) Expression of a several markers highlighting some of the differences between the i*n vivo* and the *in vitro* system. Data are represented as the mean ± SD. (G) Contact probability in logarithmic bins. Lines - mean values from biological replicates; semi-transparent ribbons - SEM. (H) Quantification of the contact enrichment in ncx_CN between distal, intra-TAD pairs of regions bound by the indicated combination of transcription factors. Data are represented as a scatter dot plot showing the mean ± SD. (I) Hi-C contact maps showing the same region as in Figure 6G but using the *in vitro* generated NPCs and CNs. (J–M) Quantification of the contact strength between pairs of Nanog (J), Pax6 (K), NeuroD2 (L) and Tbr1 (M) bound sites using the *in vitro* differentiation system. Data are represented as a scatter dot plot showing the mean ± SD. (N–Q) Contact enrichment represented between pairs of Nanog (N), Pax6 (O), NeuroD2 (P) and Tbr1 (Q) represented as the log ratio between observed and expected contacts as a function of the genomic distances. Data were smoothed using loess regression. Lines represent the mean values from biological replicates; semi-transparent ribbons show SEM.

In ESs, Nanog and Oct4 have been proposed to bring distant chromatin loci together in close proximity (de Wit et al., 2013). Using aggregate Hi-C maps, we confirmed that Nanog-bound sites interact above background in ESs. These interactions were weaker compared to Polycomb, and surprisingly, the pattern was more pronounced at longer distances (Figures S7A, S7J, and S7N).

**Figure 6:**
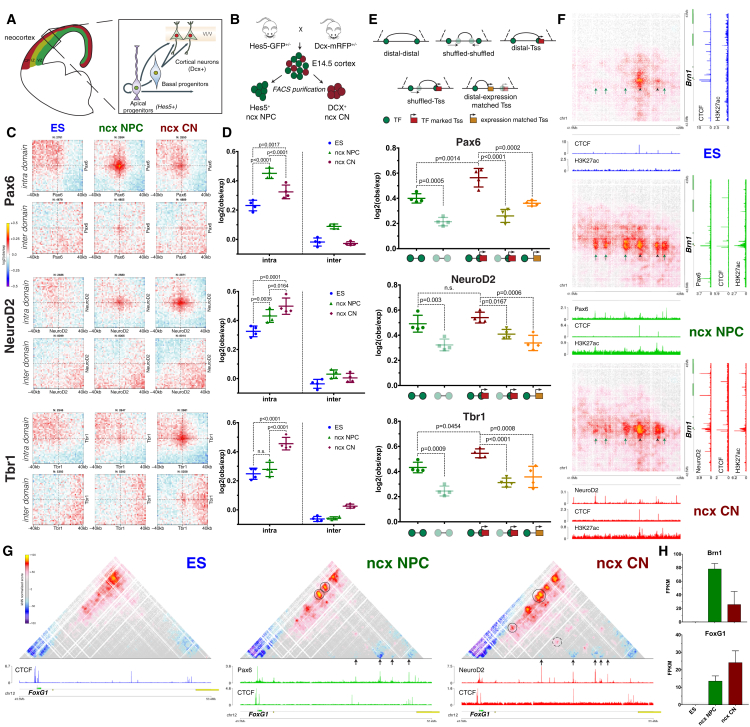
Dynamic Chromatin Contacts around Neural Transcriptional Factors *In Vivo* (A) Schematic representation of the embryonic neocortex at E14.5. The three major cell types are shown. CP: cortical plate, IZ: intermediate zone, VZ: ventricular zone. (B) Schematic representation of the fluorescence-activated-cell-sorting (FACS)-based approach to purify NPCs or CNs from the neocortex *in vivo*. (C and D) Aggregate Hi-C maps and quantifications between pairs of transcription-factor (Pax6, NeuroD2, and Tbr1)- bound sites. Data are presented as a scatter dot plot showing the mean ± SD. Statistical significance is calculated using two-way ANOVA with Tukey’s test. (E) Schematic representation of the pairs of loci queried and quantification of the contact enrichment in the either ncx NPC (for Pax6) or ncx CN (NeuroD2 and Tbr1). Data are represented as a scatter dot plot showing the mean ± SD. Statistical significance is calculated using one-way ANOVA with Tukey’s test. (F) Hi-C contact maps showing interaction between *Brn1* promoter and several Pax6 binding sites (green arrows) and contacts between CTCF sites (black arrowheads). (G) Example of a dynamic chromatin loops established between TF-bound sites (arrows) at the FoxG1 locus. Interactions between a distal TF-bound site and FoxG1 are highlighted with a circle and cell-type specific interaction between two distal sites is shown with a dashed circle. (H) Brn1 and FoxG1 expression represented as the mean ± SD of two biological replicate RNA-seq experiments. In all panels, n refers to the number of pairs examined. See also Figures S7 and S8.

**Figure S8:**
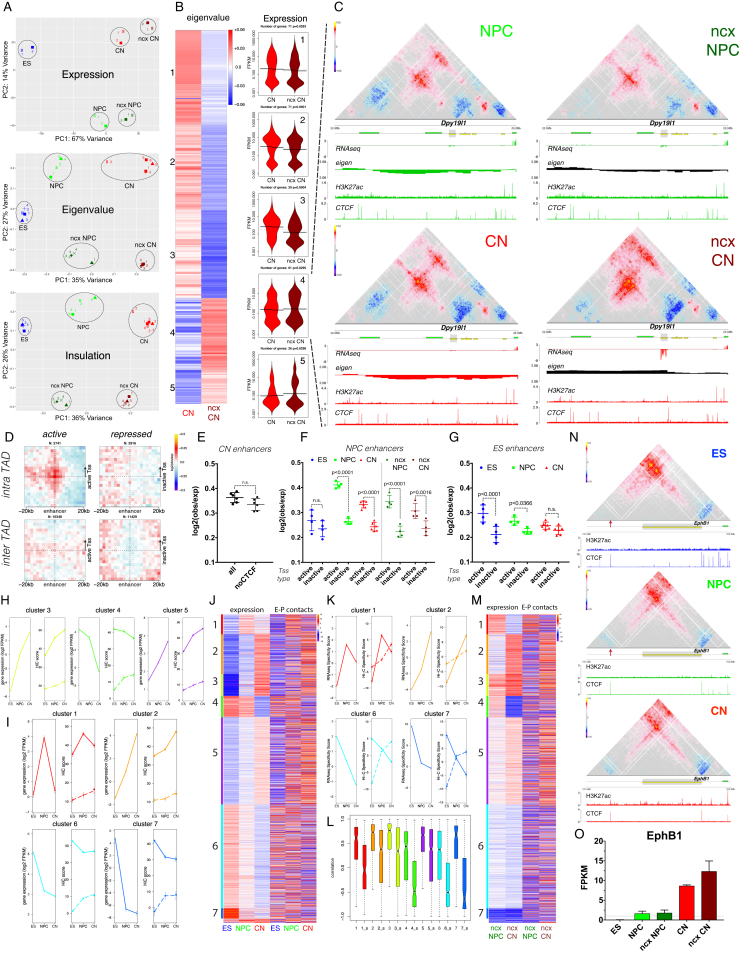
Differences between *In Vivo* and *In Vitro* Hi-C and Dynamic E-P Interactions, Related to Figures 6 and 7 (A) Principal component analysis based on gene expression, average eigenvalue in 100kb bins or average insulation score in 10kb bins. (B) K-means clustering of the eigenvalue at regions which change compartment between *in vitro* and *in vivo*. Shown is also a beanplot representing the RNA expression of genes in a cluster. Each half of the bean represents a separate RNaseq replicate. Lines show the mean value per replicate. (C) Example of a locus, which switches between B and A compartments and becomes highly expressed only in the *in vivo* cortical neurons. (D) Aggregate Hi-C maps between pairs of enhancers and either active or inactive promoters identified in CNs divided into two groups: intra domain (pairs lie inside TADs and are separated by more than 50Kb and less than 2Mb) and inter domain (pairs lie between TADs but are separated by the same distance as before). Genes were oriented according the direction of transcription. (E) Quantification of the contact strength of intraTAD pairs between CN enhancers (all or filtered for the presence of a CTCF binding site within ± 5Kb) and active CN promoters. (F) Quantification of the contact strength of intraTAD pairs between NPC enhancers and either NPC active or repressed promoters. Data are represented as a scatter dot plot showing the mean ± SD. Statistical significance is calculated using two-way ANOVA with Šídák’s multiple comparison correction. (G) As in (F) but considering pairs of ES enhancers and ES active or repressed promoters. (H) Average expression or average enhancer-promoter Hi-C score within the specified cluster (from Figure 7C). Shown are also the average Hi-C scores when enhancers were randomly shuffled within the same TAD. Error bars indicate ± SEM. (I) Same as in (H) but based on *in vivo* data. (J) Heatmap showing gene specificity scores (STAR Methods) based on either gene expression or the average enhancer-promoter interactions (per gene) *in vitro*. Clusters are the same as in Figure 7C. (K) Average gene specificity scores based on either expression or Hi-C contacts within the specified cluster. Shown are also the average Hi-C scores when enhancers were randomly shuffled within the same TAD. Error bars indicate ± SEM. (L) Boxplots representing the Pearson’s correlation between expression and contacts-based gene specificity score within a cluster. (M) Same as in (J) but based on the *in vivo* data. (N) Hi-C contact maps showing ∼2.5Mb region around *EphB1* promoter. The location of a putative CN specific enhancer is shown by red arrow. Note that this coincides with a conserved CTCF-binding site occupied in all three cell types. (O) *EphB1* expression, represented as the mean ± SD from two biological replicates.

Next, we turned our attention to neural-specific TFs. However, very few public chromatin immunoprecipitation sequencing (ChIP-seq) profiles exist, and most of those have been performed in the developing mouse neocortex (Bayam et al., 2015, Notwell et al., 2016, Sun et al., 2015). Furthermore, although the *in vitro* system we employed recapitulates some aspects of cortical development, other features, such as neuronal migration and morphogen signaling, are not. Therefore, we decided to purify NPC or CN populations directly from the mouse neocortex *in vivo*, hereafter called ncx NPC and ncx CN, respectively (Figures 6A, 6B, S7B, and S7C). We performed RNA-seq and verified our approach using known markers for neural progenitors (*Nestin*) and differentiating neurons (*Dcx*) (Figure S7D). Principal component analysis (PCA) also confirmed that NPC populations, either *in vitro* or *in vivo*, are highly similar to each other and are distinct from CNs and vice versa (Figure S8A).

We performed Hi-C on the purified NPC and CN populations *in vivo* and obtained a total of ∼3 billion uniquely aligned contacts per cell type (Table S1). Pearson correlation coefficient (Figure S7E), comparison of the eigenvector values, insulation score, and contact probability (Figures S7G and S8A) confirmed the high quality of the data and that, importantly, samples group together based on cell type. Nevertheless, we observed some important differences between the two systems. Some well-known neuronal TFs such as *Eomes* (marker for basal progenitors), *Neurog2*, *Pax6*, *NeuroD2*, and *Tbr1* were expressed in the appropriate cell type more strongly *in vivo* than *in vitro* (Figure S7F). Moreover, we identified regions that switch compartments between the *in vitro* and the *in vivo* systems, which were accompanied with corresponding changes in gene expression (Figures S8B and S8C).

*Pax6* is one of the few “master regulator” neural TFs with a well-known role in cortical development (Manuel et al., 2015). We observed a cell-type specific contact enrichment between Pax6-bound sites strongest in ncx NPC where Pax6 is highly expressed (Figures 6C and 6D). Interestingly, in contrast to Nanog or Polycomb-mediated interactions, Pax6 contacts were mostly confined within TAD boundaries (Figures 6C, 6D, and S7O). We observed a similar—albeit less strong—trend in the *in vitro* differentiated cells (Figure S7K), consistent with the lower Pax6 expression levels in NPCs *in vitro* (Figure S7F).

In order to verify these findings, we examined a ∼0.5-Mb region interacting with the neural gene *Brn1*, which is expressed strongly in NPCs *in vivo* (Figures 6G and 6H). The promoter of *Brn1* is bound by Pax6 in ncx NPC, and there are multiple H3K27ac-marked Pax6 binding sites within *Brn1* TAD (Figure 6F). Hi-C analysis identified conserved, presumably CTCF-mediated contacts (Figure 6F, arrowheads) present in all cell types and several NPC-specific contacts precisely located at Pax6-bound sites (Figure 6F, green arrows).

Next, we examined two other neuronal TFs: NeuroD2 (Bayam et al., 2015) and Tbr1 (Notwell et al., 2016). NeuroD2 and Tbr1 interactions were also mostly constrained inside TADs (Figures 6C, 6D, S7P, and S7Q) but increased during differentiation and were the strongest in CNs *in vivo*. A similar but weaker trend was observed in the *in vitro* system (Figures S7L and S7M), once again consistent with the lower expression levels *in vitro* (Figure S7F).

Finally, we asked if the interactions between TF-bound loci change because of transcriptional activation or because of “homotypic” interactions between loci bound by the same factor. We found that the contact enrichment is highest between a distal site and a Tss when they are both bound by the same TF (Figures 6E and S7H). This dynamic organization of chromatin contacts around neural TFs is evident at the *FoxG1* locus, where cell-type specific interactions were organized around Pax6 and NeuroD2 binding sites (Figure 6G), even though FoxG1 was expressed in both NPCs and CNs (Figure 6H).

Collectively, these data show that dynamic chromatin interactions are much more prevalent than previously anticipated. They are frequently related to cell-type specific TFs, which contribute to a distinct chromosome folding. TADs appear to constrain the interactions between some, but not all, TFs, and even inside a TAD, contacts are preferentially established between a distal site and a Tss, both bound by the same TF. Finally, optimal genomic distance for a TF-associated chromatin interaction can vary, suggesting that specific biophysical features of TF function lead to distinct contributions to global chromosome folding.

### Enhancer-Promoter Contacts Are Mostly Cell-Type Specific and Are Generally Established Concomitantly with Gene Expression

**Figure 7:**
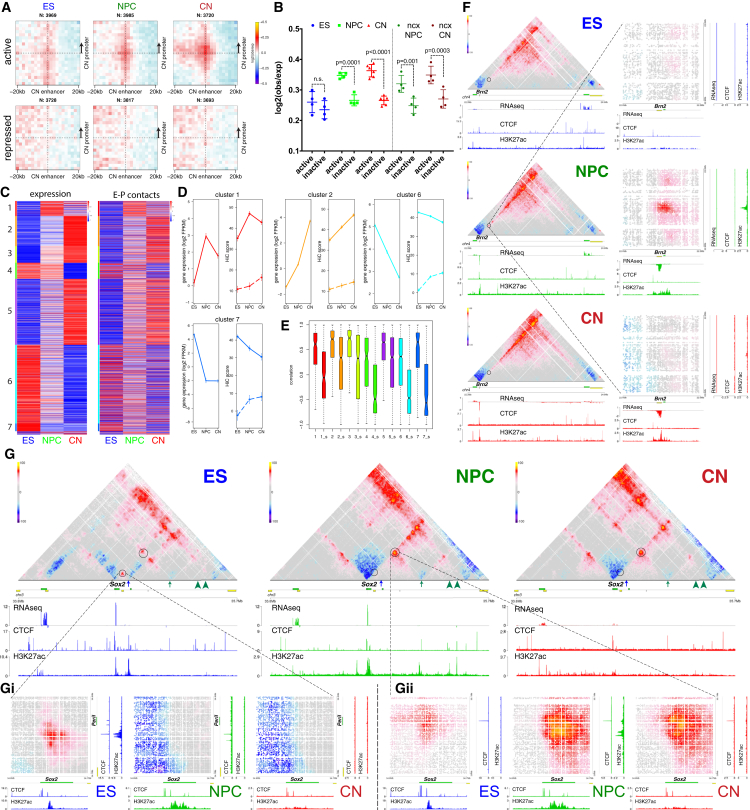
Enhancer-Promoter Contacts Are Mostly Cell-Type Specific and Are Correlated with Gene Expression (A and B) Aggregate Hi-C maps and quantification between intra-TAD pairs of enhancers and either active or inactive promoters identified in CNs. Genes were oriented according to the direction of transcription. Data are represented as a scatter dot plot showing the mean ± SD. Statistical significance is calculated using two-way ANOVA with Šídák correction. (C) Heat map showing *Z* scores based on either gene expression (fragments per kb of transcript per million mapped reads [FPKM]) or the average E-P interactions per gene. Genes are partitioned using k-means clustering on the RNA expression data across all cell types. (D) Average expression or average E-P Hi-C score within the specified cluster. Shown are also the average Hi-C scores when the enhancer contact anchor was randomly shuffled within the same TAD (dashed lines). Error bars indicate ± SEM. (E) Boxplots representing the Pearson correlation coefficient between expression and average E-P interactions per gene, either real or shuffled (shuffled are indicated by the “_s” prefix). (F) Hi-C contact maps showing ∼2.6-Mb region around *Brn2* promoter. Insets show a magnified view of the contact between the *Brn2* promoter and an NPC-specific enhancer (dashed circle). (G) Hi-C contact maps showing ∼2-Mb region at the *Sox2* locus. The positions of two putative NPC-specific enhancers are indicated by green arrowheads. Insets showing the interaction between Sox2 promoter and an ES-specific enhancer (blue arrow, Gi); or Sox2 promoter and a known NPC-specific enhancer (green arrow, Gii). See also Figure S8 and Table S3.

Enhancer-promoter (E-P) contacts have been difficult to observe in Hi-C, and it is unclear how strong they are compared to the surrounding regions. Using aggregate Hi-C maps, we observed enrichment only between pairs of enhancers and active promoters, but not when we examined repressed genes (Figure S8D). Importantly, pairwise E-P interactions were constrained primarily inside TADs (Figure S8D), were gradually increased during differentiation *in vitro* and *in vivo* for CN-based pairs (Figures 7A and 7B), and were not strongly dependent on CTCF binding (Figure S8E). Furthermore, intra-TAD-E-P interactions were strongest in their respective cell type (Figures S8F and S8G and Table S3).

These results suggest that E-P interactions are highly dynamic and cell-type specific. To directly address this hypothesis, we clustered highly variable genes based on expression and found that the change in average expression is highly correlated with the change in average E-P interaction score within a cluster (Figures 7C–7E). Furthermore, the average Hi-C scores and the correlation between expression and chromatin contacts were strongly decreased when we randomly shuffled the position of the enhancer region within the same TAD (Figures 7D, 7E, and S8H). Analogous results were obtained *in vivo* (Figure S8I). In order to confirm these findings, we also used the “gene specificity score” as described previously (Javierre et al., 2016). We observed high correlation between scores based either on gene expression or on average E-P Hi-C score per gene, both *in vitro* (Figures S8J–S8L) and *in vivo* (Figure S8M).

Next, we examined selected differentially expressed genes. We identified a NPC-specific enhancer, which interacted strongly with the promoter of the neuronal TF *Brn2* gene only in NPCs, and this interaction was disrupted in CNs when the enhancer was inactive (Figure 7F).

*Sox2* interacts with a super-enhancer in ESs (Li et al., 2014) and has been suggested to undergo an enhancer switch between ESs and NPCs based on 5C data (Phillips-Cremins et al., 2013). We observed a strong decrease in the interactions between *Sox2* promoter and the ES-specific enhancer (Figure 7G, blue arrow) during differentiation, which coincides with a decrease in both CTCF binding and the H3K27ac mark on the enhancer (Figure 7Gi). Conversely, the contact between Sox2 promoter and the NPC-specific enhancer (Figure 7G, green arrow) increased in NPCs. Importantly, our high resolution allowed us to discriminate between a conserved CTCF-mediated contact located less than 20 kb away from the enhancer and the E-P interaction, which is evident only in NPCs when the enhancer is active (Figure 7Gii). We also identified two putative NPC-specific Sox2 enhancers (Figure 7G, green arrowheads), which independently gained H3K27ac and contacted Sox2 promoter primarily in NPCs (Figure 7G).

Collectively, the data show that E-P interactions are primarily intra-TAD and are dynamically regulated during development. Although we did identify instances of pre-set chromatin interactions between enhancers and their cognate target genes that precede transcriptional activation (Figures 7C, S8N, and S8O), the majority of those interactions appear to be established concomitantly to gene expression and are also disrupted when the genes become repressed. This suggests that regulating the contact probability between an enhancer and a promoter may represent an important mechanism in the control of gene expression and cell fate.

## Discussion

The analysis of a well-controlled differentiation system and its *in vivo* cell counterparts shows that cell differentiation is accompanied by a global chromatin reorganization and a change in compartmentalization with a decrease in long-range interactions between A-type domains concomitant with an increase in contacts between inactive B-type domains. Previous studies have shown that heterochromatin regions aggregate in the center of the nucleus in retinal neurons (Solovei et al., 2013) or that olfactory receptor genes interact strongly in postmitotic neurons (Clowney et al., 2012). Importantly, in both cases, a reduction in the levels of lamin B receptor was observed, which is also evident in our differentiation system (Figure S1K), suggesting a common mechanism.

In addition, the high spatial resolution achieved allowed disentangling the roles of different, closely located chromatin components in insulation and long-range contacts. We found transcription to be highly correlated with local chromatin insulation. Therefore, although we confirmed that most TAD boundaries are conserved (Dixon et al., 2012), novel borders can occur at promoters of developmentally regulated genes. Furthermore, the correlation between transcription and insulation also extends within TADs. However, we show that activating transcription is not sufficient to cause chromatin insulation, and thus, other factors such as E-P interactions and specific TFs likely contribute to creating insulation. Alternatively, changes in chromatin conformation precede and may enable gene expression at specific loci. These findings complement recent results in *Drosophila* development, which suggested that transcription is not necessary for boundary formation (Hug et al., 2017).

We show that, consistent with previous findings (Rao et al., 2014), CTCF sites interact predominantly in the convergent orientation; however, the strength of this type of contact increases during differentiation. This is somewhat counterintuitive, as the absolute levels of CTCF binding actually decrease during differentiation. An alternative explanation might be that cohesin binding and potentially the processivity of the loop-extruding complex might be affected. Consistent with this hypothesis, we find that expression of cohesin unloading factors such as Wapl and Pds5a is decreased during neural differentiation (Figure S4J), and the increase in looping strength and contact frequency upon Wapl removal in human cells (Haarhuis et al., 2017) correlates well with this.

In addition to CTCF and promoters, we showed that different types of chromatin contact behaviors and loops coexist in different cell types. Surprisingly, we find that the bodies of highly expressed genes interact strongly both in *cis* and in *trans* to form clusters of loops. These interactions are strongly correlated with the number of splicing events per gene with the strongest contacts occurring between genes that undergo most splicing. Splicing foci have been observed in live cells (Rino et al., 2007), but whether the contacts we observed are directly linked to co-transcriptional splicing remains to be seen.

In ESs, the strong long-range interactions also involve Polycomb-bound genes and, to a lesser extent, genes bound by pluripotency TFs. These networks are reorganized in neuronal differentiation, which in the case of Polycomb appears to correlate with PRC1 binding levels.

Within the neural lineage, we observed increased contact strength between loci bound by distinct TFs. These interactions are cell-type specific and point to a potential novel role of these TFs in cortical development. These contacts do not appear to be just a consequence of transcription activation, but it remains to be seen if they truly represent homotypic interactions. In that regard, Pax6 has been shown to directly interact with members of the BAF chromatin remodeling complex (also known as mSWI/SNF) in adult neural progenitors (Ninkovic et al., 2013), but whether and how that contributes to chromatin architecture is unknown.

Finally, E-P interactions are highly dynamic and are usually established concomitantly with gene expression. The high resolution of our Hi-C approach allowed us to determine the contact strength of the E-P interactions relative to the surrounding regions while controlling for global changes such as compaction. Our observations support a model where dynamic chromatin looping from enhancer regions triggers gene activation (Deng et al., 2014), although clear violations of this principle exist, as previously seen in *Drosophila*, where many E-P loops are pre-established (Ghavi-Helm et al., 2014).

This work provides a comprehensive view of chromatin organization. It shows that different regulatory factors establish preferential contacts at different scales. These range from close cis interactions such as promoter-gene body; to long-range TAD-delimited contacts such as those between enhancers and promoters and TF binding sites; and finally, to very long-range contacts involving promoters, Polycomb, heterochromatin regions, and a subset of TF binding sites. This comprehensive dataset thus provides a rich resource to study dynamic chromatin interactions involving different regulatory players of cell fate and differentiation. From a developmental perspective, ultra-deep Hi-C maps from purified neocortical cell populations will additionally allow for a deeper understanding of how genome architecture is related to function *in vivo*.

## STAR★Methods

### Key Resources Table

**Table:** 

REAGENT or RESOURCE	SOURCE	IDENTIFIER
**Antibodies**		

Pax6, rabbit, polyclonal	Covance	Cat# PRB-278P-0100
Tuj1, rabbit, polyclonal	Sigma-Aldrich	Cat# T2200; RRID: AB_262133
GFP, rabbit, polyclonal	ThermoFisher	Cat# A10262; RRID: AB_2534023
H3K4me1, rabbit, polyclonal	Active Motif	Cat# 39297; RRID: AB_2615075
H3K4me3, rabbit, monoclonal	Millipore	Cat# 04-745; RRID: AB_1163444
H3K9me3, rabbit, polyclonal	Abcam	Cat# ab8898; RRID: AB_306848
H3K27me3, rabbit,	Active Motif	Cat# 39155; RRID: AB_2561020
H3K36me3, rabbit, polyclonal	Abcam	Cat# ab9050; RRID: AB_306966
CTCF, rabbit, monoclonal	Cell Signaling	Cat# 3418S; RRID: AB_2086791
Ring1B, rabbit, monoclonal	Cell Signaling	Cat# 5694P; RRID: AB_10706357

**Chemicals, Peptides, and Recombinant Proteins**		

Leukemia Inhibitory Factor (LIF)	Millipore	ESG1106
Cyclopamine	Merck	239803
B-27 without retinoic acid	ThermoFisher	12587-010
N-2	ThermoFisher	17502-048
Fetal Bovine Serum, ES-qualified	ThermoFisher	16141-079
BSA Fraction V	ThermoFisher	15260-037

**Critical Commercial Assays**		

FxCycle, Far Red	ThermoFisher	F10348
TruSeq ChIP Sample Preparation Kit	Illumina	IP-202-1012
TruSeq Stranded Total RNA Library Kit	Illumina	RS-122-2301

**Deposited Data**		

Raw Hi-C data for the 3 in vitro, 2 in vivo generated cell-types and CRISPR-dCas9 experiments	This study	GEO: GSE96107
CTCF ChIP-seq in sorted Oct-GFP+ ESs	This study	GEO: GSE96107
CTCF ChIP-seq in sorted Sox1-GFP+ NPC cells	This study	GEO: GSE96107
CTCF ChIP-seq in sorted Tau-GFP+ CN cells	This study	GEO: GSE96107
Ring1B ChIP-seq in sorted Oct-GFP+ ESs	This study	GEO: GSE96107
Ring1B ChIP-seq in sorted Sox1-GFP+ NPC cells	This study	GEO: GSE96107
Ring1B ChIP-seq in sorted Tau-GFP+ CN cells	This study	GEO: GSE96107
H3K4me1 ChIP-seq in sorted Sox1-GFP+ NPC cells	This study	GEO: GSE96107
H3K4me1 ChIP-seq in sorted Tau-GFP+ CN cells	This study	GEO: GSE96107
H3K4me3 ChIP-seq in sorted Sox1-GFP+ NPC cells	This study	GEO: GSE96107
H3K4me3 ChIP-seq in sorted Tau-GFP+ CN cells	This study	GEO: GSE96107
H3K9me3 ChIP-seq in sorted Sox1-GFP+ NPC cells	This study	GEO: GSE96107
H3K9me3 ChIP-seq in sorted Tau-GFP+ CN cells	This study	GEO: GSE96107
H3K27ac ChIP-seq in sorted Sox1-GFP+ NPC cells	This study	GEO: GSE96107
H3K27ac ChIP-seq in sorted Tau-GFP+ CN cells	This study	GEO: GSE96107
H3K27me3 ChIP-seq in sorted Sox1-GFP+ NPC cells	This study	GEO: GSE96107
H3K27me3 ChIP-seq in sorted Tau-GFP+ CN cells	This study	GEO: GSE96107
H3K36me3 ChIP-seq in sorted Sox1-GFP+ NPC cells	This study	GEO: GSE96107
H3K36me3 ChIP-seq in sorted Tau-GFP+ CN cells	This study	GEO: GSE96107
Strand specific total RNA from sorted Sox1-GFP+ NPC cells	This study	GEO: GSE96107
Strand specific total RNA from sorted Tau-GFP+ CN cells	This study	GEO: GSE96107
Strand specific total RNA from sorted Hes5-GFP+/Dcx-mRFP- ncx_NPC cells	This study	GEO: GSE96107
Strand specific total RNA from sorted Hes5-GFP-/Dcx-mRFP+ ncx_NPC cells	This study	GEO: GSE96107

**Datasets Reanalyzed**		

H3K4me1 in ESs (E14)	ENCODE	ENCSR032JUI
H3K4me3 in ESs (E14)	ENCODE	ENCSR000CGO
H3K9me3 in ESs (E14)	ENCODE	ENCSR857MYS
H3K27ac in ESs (E14)	ENCODE	ENCSR000CGQ
H3K27me3 in ESs (E14)	ENCODE	ENCSR059MBO
H3K36me3 in ESs (E14)	ENCODE	ENCSR253QPK
Nanog in ESs (E14)	ENCODE	ENCSR779CZG
Smc1 in ESs (v6.5)	(Kagey et al., 2010)	GEO: GSE22562
Pax6 in E12.5 embryonic forebrain	(Sun et al., 2015)	GEO: GSE66961
NeuroD2 in E14.5 neocortex	(Bayam et al., 2015)	GEO: GSE67539
Tbr1 in E15.5 neocortex	(Notwell et al., 2016)	GEO: GSE71384
Replication timing – ES	(Hiratani et al., 2010)	GEO: GSM450272
Lamin B1 DamID - ES	(Peric-Hupkes et al., 2010)	GEO: GSE17051

**Experimental Models: Cell Lines**		

E14TG2a	https://www.lgcstandards-atcc.org/Products/All/CRL-1821.aspx?geo_country=us	N/A
Oct4-GFP (Oct4GiP)	(Ying et al., 2003)	N/A
Sox1-GFP (46C)	(Ying et al., 2003)	N/A
Tau-GFP (J1 background)	(Bibel et al., 2004)	N/A

**Experimental Models: Organisms/Strains**		

Mouse: Hes5-GFP (C57/BL6 background)	(Basak and Taylor, 2007)	N/A
Mouse: Dcx-mRFP (C57BL/6J-Tg(Dcx-mRFP)15Qlu/J)	The Jackson Laboratory	JAX: 024905

**Software and Algorithms**		

Shaman R package for a-parametric Hi-C normalization	N/A	https://bitbucket.org/tanaylab/shaman
FlowJo	N/A	www.flowjo.com/
imageJ	N/A	https://imagej.nih.gov/ij
Bowtie2 v2.2.0	N/A	http://bowtie-bio.sourceforge.net/bowtie2/index.shtml
Samtools v0.1.19	N/A	http://samtools.sourceforge.net/
DeepTools2	N/A	http://deeptools.readthedocs.io/en/latest/
AQUAS ChIP-seq processing pipeline	N/A	https://github.com/kundajelab/chipseq_pipeline
CSAW	N/A	bioconductor
STAR v2.5	N/A	https://github.com/alexdobin/STAR
DESeq2	N/A	https://bioconductor.org/packages/release/bioc/html/DESeq2.html
C-world (Hi-C analysis software)	Job Dekker lab	https://github.com/dekkerlab/cworld-dekker
GraphPad Prism	N/A	https://www.graphpad.com/

### Contact for Reagent and Resource Sharing

Further information and requests for resources and reagents should be directed to and will be fulfilled by the Lead Contact, Giacomo Cavalli (giacomo.cavalli@igh.cnrs.fr).

### Experimental Model and Subject Details

#### Cell Lines

E14Tg2a, Oct4-GFP and Sox1-GFP feeder-free or Tau-GFP (grown on irradiated CF-1 MEF feeders (Tebu-Bio, Cat.N: GSC-6001G)) cell lines were maintained as described in (Gaspard et al., 2008). In brief, cells were cultured in DMEM (ThermoFished, Cat.N:21969-035), suppelemented with 15% FBS (ES-qualified, ThermoFished, Cat.N:16141-079) 1,000 U/mL of LIF (Millipore, Cat.N: ESG1106), 0.1 mM of non-essential amino acids (ThermoFished, Cat.N: 11140-035), 1 mM of glutamax (ThermoFisher, Cat.N: 35050-038), 50U of penicillin and streptomycin (ThermoFirsher, Cat.N: 15070-063) and 0.1 mM of 2-mercaptoethanol (ThermoFisher, Cat.N: 31350-010). Media was changed every day and cells were passaged every two days using StemPro Accutase (ThermoFisher, Cat.N: A11105-01).

Cells were differentiated into neuronal progenitors and cortical neurons as described in (Gaspard et. al., 2008) with minor changes. In brief, cells were plated at low density (2.5x10^5^ cells per plate) on gelatin-coated 10cm dishes in ES media and after 12h cultured in DDM media (DMEM/F12 + GlutaMAX (ThermoFisher, Cat.N: 31331-028), supplemented with 1x N2 (ThermoFisher, Cat.N: 17502-048), 1 mM of sodium pyruvate (ThermoFisher, Cat.N: 11360-070), 500 ug/ml BSA Fraction V (ThermoFisher, Cat.N:15260-037), 0.1mM of 2-mercaptoethanol and 50U of penicillin and streptomycin) for a total of 12 days. Cyclopamine (Merck – 239803) was added from day 2 to day 10 of differentiation. Media was changed every two days. After 12 days of differentiation, neural progenitors were dissociated using StemPro Accutase, plated on poly-lysine (Sigma, Cat.N: P2636) /laminin (Sigma, Cat.N: 11243217001) and cultured in 1:1 mixture of DDM and Neurobasal/N27 media (ThermoFisher, Cat.N: 21103-049, supplemented with 1x B27 without retinoic acid (ThermoFisher, Cat.N: 12587-010) and 2mM GlutaMax) for either additional 2 days (D12+2) to obtain neural progenitors (NPC), or additional 9 days (D12+9) to obtain cortical neurons (CNs).

#### Generation of CRISPR-dCas9 Lines

E14tg2a cells were infected by a lentivirus contain dCas9-VP64 (Addgene, Cat.N 61425) or empty dCas9 (generating by removing the VP64 from the dCas9-VP64). Four days after infection cells were selected by adding 15ug/ml of basticidine (GIBCO, Cat.N A11139-03) during one week. To generate dCas9-VP64/p65-Hsf1 cell line, dCas9-VP64 positive cells were then additionally infected by lentivirus contain MS2-P65-HSF1 helper protein (Addgene, Cat.N 61426). Four days after infection positive cells were selected by adding hygromycine at 1mg/ml (GIBCO, Cat.N 10687010).

Guide RNAs (Zfp604 – AGAAAGCGGAATGAGAAGTT and Sox4 - TTGCTCTGTAAATTGGAATG) were designed and cloned in lenti sgRNA(MS2) zeo backbone (Addgene, Cat.N 61427) according to Zhang lab protocol (http://sam.genome-engineering.org/). The above mentioned CRISPR-dCas9 cell lines (plus the original E14Tg2a cell line) was infected by the sgRNA lentivirus and selected with 250ug/ml of zeocin (Invitrogen Cat.N R250-01) for 5 days. The first biological Hi-C/RNA replicate was collected one week after the original infection date, while the second was done one passage later (9 days post-infection).

#### Mouse Strains

Hes5-GFP mice were a kind gift from Verdon Taylor. Dcx-mRFP (C57BL/6J-Tg(Dcx-mRFP)15Qlu/J) mice were obtained from the Jackson Laboratory. The transgenic lines were housed and maintained following the guidelines of the Animal Care and Use Committee of the Institut National de la Santé et de la Recherche Médicale (INSERM) in accordance with the European Council directive (2010/63/UE) for the protection and use of vertebrate animals. To purify *in vivo* cortical progenitors and differentiating neurons, heterozygous parents (C57BL/6J-Hes5-GFP^+/−^ and C57BL/6J-Dcx-mRFP^+/−^) were bred, at E14.5 embryos were harvested, examined using whole mount fluorescence and the brains of double-positive (GFP^+^/RFP^+^) littermates were dissected. The somatosensory neocortex was placed in DMEM/F-12 media, mechanically dissociated using P1000 tip and further incubated for 2 min with StemPro Accutase at 37C. 3x volume of DMEM/F12 was then added, cells were transferred to cold 1xPBS + 2% FBS, and immediately processed for FACS. Each biological replicate represents a separate mating with 1-3 pooled littermates.

### Method Details

#### Cell Isolation and Purification for HiC

For the *in vitro* differentiation system, cells were dissociated using StemPro Accutase (for ESs and NPCs) or 0.05% Trypsin-EDTA (ThermoFisher, Cat.N: 25300-054) (for CNs). After fixation for 10 min at RT with freshly prepared 1% formaldehyde in PBS (ThermoFisher, Cat.N: 28908), the reaction was quenched by adding 0.2M glycine solution for 5min. Cells were permeabilized using 0.1% saponin and concentration was adjusted to 1x10^6^ cells / mL PBS. 1 μL/mL of fxCycle far red dye (ThermoFisher, Cat.N: F10348) and 5 μL/mL RNase A (20mg/mL) were added and samples were incubated for 30min at room temperature protected from light with slight agitation. After washing once with cold 1xPBS, samples were resuspended in cold 0.5% BSA in PBS at a concentration of ∼10^7^ cells/mL and immediately processed for FACS. FACS was performed using BD FACSAria and appropriate gates were set based on the relative levels of GFP and fxCycle as described in Figure S1 in order to isolate GFP^+^ / G_0_G_1_ cells.

For *in vivo* purified cells, between 250 000 and 600 000 cells per replicate were FACS-purified directly after dissociation and then immediately fixed and quenched as described, as fixation first quenched mRFP signal completely (data not shown). The appropriate gates to isolate GFP^+^/RFP^-^ and RFP^-^/GFP^+^ cells were set as described in Figure S7B.

After FACS sorting, cell pellets were frozen in liquid nitrogen and stored at −80C.

#### HiC Library Preparation

HiC and library preparation was carried out using the *in situ* method as described previously (Rao et al., 2014) with minor modifications. In order to maximize library complexity, FACS-purified samples were split into batches of 1x10^6^ cells and processed separately. In brief, cells were digested overnight at 37C using 500U of DpnII. After biotin filling, proximity ligation was carried out for 4 hours at 18C with 2000U T4 DNA Ligase. After reverse-crosslinking, DNA was purified using ethanol precipitation and sheared to 300-400bp fragments using Covaris S220 sonicator. Ligation fragments containing biotin were immobilized on MyOne Streptavidin T1 beads (ThermoFished Cat.N: 65602), end-repaired and a-tailed as described. NEXTflex adaptors (Bioo Scientific, Cat.N: 514101) were then ligated and fragments were PCR amplified using KAPA HiFi Library Ampification Kit (Kapa Biosystems, Cat.N: KK2620) for 6-8 cycles. DNA was then double-size selected using AMPure XP beads (Agencourt, Cat.N: A63881) in order to isolate fragments between 300 and 800bp.

#### Strand-Specific Total RNA Library Preparation

Cells were processed for FACS as described for HiC, except without fixation, permeabilization or fxCycle addition. GFP^+^ purified cells were immediately pelleted and resuspended in TRIzol (ThermoFisher, Cat.N: 15596026). After 5min at room temperature samples were vortexed for 20 s, 0.2x volumes of chloroform was added, tubes were mixed by inverting and samples were centrifuged at 13,000 rpm at 4C for 15min. The aqueous phase was then processed using the RNeasy Mini Kit (QIAGEN, Cat.N: 74104) with DNase treatment, according to the manufacturer’s instructions. Purified RNA (maximum of 1ug) was then used for library generation using the TruSeq Stranded Total RNA Library Kit according to the manufacturer’s instructions.

#### ChIP and Library Preparation

Cells were processed for FACS as described for HiC, except for permeabilization or fxCycle addition. GFP^+^ purified cells were immediately pelleted, frozen in liquid nitrogen and stored at −80C until further use. 1x10^6^ cells per IP for chromatin marks, or 2.5x10^6^ cells for CTCF/Ring1B were then thawed on ice, resuspended in cold cell lysis buffer (10mM Tris pH 8, 10mM NaCl, 0.2% NP-40) + 1xEDTA-free Protease Inhibitors. 10mM sodium butyrate was added if H3K27ac was ChIPed. Cells were lysed for 20min, washed once with cold PBS and resuspended in 50ul per IP cold nuclei lysis buffer (50mM Tris pH8, 10mM EDTA, 1% SDS) + 1xProtease Inhibitors / Sodium Butyrate. Nuclei were lysed for 10min at 4C with rotation and then sonicated for 16-18 cycles using Bioruptor (Diagenode). After sonication, 5x volumes of IP dilution buffer (20mM Tris pH8, 2mM EDTA, 150mM NaCl, 1% Triton X-100, 0.01% SDS + protease inhibitors) was added, chromatin was precleared using 25ul Protein A dynabeads (ThermoFisher, Cat.N: 10002D) / 1mL for 1hrs at 4C with rotation. Meanwhile, 25ul beads / IP were washed once with cold 0.5% BSA in PBS, and incubated with the antibody for 4-5hrs at 4C in 0.5ml 0.5% BSA in PBS). Beads were then washed once with 0.5% BSA in PBS, added to the precleared chromatin and incubated overnight at 4C with rotation. Beads were then washed once with cold IP wash buffer 1 (20mM Tris pH8, 2mM EDTA, 50mM NaCl, 1% Triton X-100, 0.1% SDS), twice with high salt wash buffer (20mM Tris pH8, 2mM EDTA, 500mM NaCl, 1% Triton X-100,0.1% SDS), once with cold IP wash buffer 2 (10mM Tris pH8,1mM EDTA,250mM LiCl,1% NP-40,1% sodium deoxycholate) and twice with cold TE buffer (1mM Tris pH8, 1mM EDTA). DNA:protein complexes were then eluted twice for 15min at 65C in 100ul elution buffer (100mM NaHCO_3_, 1%SDS) each time. 16ul 5M NaCl was then added and samples + inputs were reverse cross-linked at 65C, RNase A and proteinase K treated and purified using ultrapure phenol/chloroform (ThermoFisher, Cat.N: 15593-049). Libraries were prepared using Illumina’s TruSeq ChIP Sample Preparation Kit, according to the manufacturer’s instructions with two exceptions: libraries were PCR amplified using KAPA HiFi Library Ampification Kit for 10-12 cycles to maximize complexity and the gel-based size-selection was performed after PCR amplification.

#### Library QC and Sequencing

Before sequencing, libraries were quantified by qPCR and the size distribution was assessed using Agilent 2100 Bioanalyzer/ Caliper Labchip GX. Libraries were then sequenced 2x50bp paired-end run (except some ChIP-seq libraries which were sequenced at 1x50bp single-end) on the Illumina HiSeq2000/HiSeq2500 platforms at the BGI Hong Kong facility.

### Quantification and Statistical Analysis

#### ChIP-Seq Analysis

Fastq files were aligned to the mm10 reference genome, PCR duplicates were removed using Samtools and normalized genome coverage tracks were generated from uniquely mapping reads (mapq > 30) using deepTools2. In order to avoid double counting for paired-end data, reads with mates were extended to match the fragment size defined by the two read mates, and the second read mate was discarded. Single-end reads, unmated reads, mate reads that map too far apart (> 4x fragment length) were extended by 200bp. Biological replicates were pooled and coverage was then calculated as average reads per million of mapped reads (RPM) in 10bp bins.

To determine the peaks for ChIPs with narrow binding profiles (CTCF, Ring1B, H3K4me3, H3K27ac, Pax6, NeuroD2 and Tbr1), datasets were uniformly processed using the AQUAS Transcription Factor pipeline (based off the Encode (phase-3) specifications) with default parameters (IDR = 5%). Peaks for Nanog, ES_H3K4me3 and ES_H3K27ac were downloaded directly from the ENCODE website (see Key Resources Table) and used without modifications For broad histone marks, regions with strong signal enrichment (> 98% based on the global enrichment quantile using 1Kb bins) were used. ChIP enrichment is then defined as the –log2(1- global percentile rank). Differential peaks were detected using the R package Csaw.

#### RNA-Seq Analysis

Fastq files were aligned to the mm10 reference genome using STAR version 2.5.0a in basic twoPass mode using the “Encode” options as specified in the manual. PCR duplicates were removed and RPM normalized strand-specific coverage tracks based on uniquely aligned reads were also produced using STAR in “inputAlignmentsFromBAM” runMode. Reads overlapping with annotated genes (Ensembl build GRCm38.p4) were counted using the summarizeOverlaps function from the R package “GenomicAlignments” in strand-specific, paired –end mode. FPKM (Fragments per kilobase per million mapped fragments) counts and differential expression was estimated using DESeq2. Genes with FPKM > = 1 were considered expressed for subsequent analysis.

#### Hi-C Sequence Alignment and QC

Raw sequencing reads were mapped independently to the mm10 reference genome using Bowtie2 in local alignment mode. The uniquely mapped (MAPQ > 36) reads were translated into a pair of fragment-ends (fends) by associating each read with its downstream fend. PCR duplicates and reads mapping to the same restriction fragment were excluded. Library Statistics are presented in Table S1 and include total reads sequenced and number of valid interactions after filtering. Contacts separated by less than 1kb were not considered for subsequent analysis, as the vast majority of this ultra-close *cis* contacts originate from non-digested DNA.

#### Hi-C Data Processing

In order to retain maximum information and utilize the depth of the generated data, all analysis has been performed at fragment end resolution with no binning unless otherwise stated. To generate an expected model, we shuffled the observed Hi-C contacts using the Shaman R package (https://bitbucket.org/tanaylab/shaman) with default parameters. Briefly, cis-contacts were randomized using a Markov Chain Monte Carlo-like approach per chromosome (Olivares-Chauvet et al., 2016), such that the marginal coverage and contact distance distribution are preserved but any specific features, such as compartments, TADs or loops are not maintained. These expected HiC maps were generated for each biological replicate separately and contain twice the number of observed cis-contacts. We then calculated the score for each contact in the observed contact matrix using K nearest neighbors (kNN) strategy (Olivares-Chauvet et al., 2016). In brief, the distributions of two-dimensional Euclidean distances between the observed contact and its nearest 200 neighbors in the pooled observed and pooled expected (per cell type) data are compared, using Kolmogorov–Smirnov D statistics to visualize positive (higher density in observed data) and negative (lower density in observed data) enrichments. These D-scores are then used for visualization (−100 to +100 scale) and are referred to as HiC scores in the text.

#### Contact Probability

Contact probability as a function of the genomic distance was calculated as previously described (Olivares-Chauvet et al., 2016). We calculated the distribution of the Hi-C contacts either as a log10 contact probability in log10 genomic distance bins, or in order to better visualize differences between conditions, as a simple contact probability (sum of the observed counts per log2 bin, divided by the all of the observed contacts, without normalizing for the bin size). We measured the “contact probability scaling” exponent as the slope of the best-fit line of the cis-decay curve when plotted on log-log axes, within a chosen range of distances.

In addition to the genome-wide contact probability curves which were aggregated across individual chromosomes, we also generated contact probability maps where we considered only contacts that are located completely within a TAD (intra-TAD cis-decay curve) or inter-TAD. For Figure S1L we also took into consideration whether the interaction is across compartments (A-B or B-A) or within a compartment (A-A, B-B).

#### Insulation, TAD, and TAD Boundary Calling

To define insulation based on observed contacts we used the insulation score as previously defined (Nagano et al., 2017, Olivares-Chauvet et al., 2016). The insulation score was computed individually on replicates and on the pooled contact map at 1Kb resolution within a region of ± 250Kb and is multiplied by (−1) so that high insulation score represents strong insulation. In order to account for any genome-wide changes in the insulation score, we further normalized it by multiplying with a factor defined as the average insulation score across all 1Kb genomic bins in each cell type, divided by the mean of all cell types. Domain boundaries were then defined as the local 2Kb maxima in regions, where the insulation score is above the 90% quantile of the genome-wide distribution. Boundaries within 10Kb of each other were merged and only boundaries, which were detected in at least 3 biological replicates, were retained. Domains shorter than 50Kb were discarded. To identify differential boundaries we pooled boundaries detected in the respected cell types, merging together boundaries if they were located within 10Kb. We consider borders as significantly different if they fulfill the following criteria:

1. boundary insulation score is significantly different between cell types (fdr < 0.01 using the Benjamini-Hochberg adjusted t test)
2. boundary variance (defined as the maximum insulation score – minimum insulation score across conditions). We consider the top 20% based on variance, in order to avoid any potential false positives.
3. crossing a boundary threshold – it is considered as a boundary in one condition but not in another (as defined above).

#### Identification of A and B Compartments

TADs were assigned to either the A- or the B- compartment as described (Nagano et al., 2017). In brief, we used k-means clustering (K = 2) on the log2 ratio of observed and expected (based on genomic length) inter-TAD contacts in *trans*. Domains in each cluster exhibit distinct signature based on the enrichment of chromatin marks, replication timing and Lamin B1 DamID enrichment where available (Figure S1I), prompting us to assign cluster 1 to the B-, inactive compartment and cluster 2 to the A-, active compartment. In order to identify compartment transitions, the number of TAD boundaries between adjacent domains of different types (A-to-B or B-to-A transition) was divided by the total number of TAD boundaries within that cell type. To determine the compartment strength, we calculated the log2 ratio of observed versus expected contacts (intrachromosomal separated by at least 10Mb) either between domains of the same (A-A, B-B) or different type (A-B).

As another measure of compartmentalization which relies on the intra-chromosomal contacts, we calculated the dominant eigenvector of the contact matrices binned at 100Kb as described (Lieberman-Aiden et al., 2009) using scripts available at (https://github.com/dekkerlab/cworld-dekker).

#### Aggregate Insulation and Hi-C Maps

To quantify the average insulation around a set of regions based on the Hi-C contacts, we merged adjacent regions if they were separated by less than 500bp and extracted the observed and the expected contacts in a ± 200Kb window. We calculated the number of contacts (either observed or expected) binned by 500bp and normalized them by the total number of contacts (observed or expected respectively) in the window. We then plotted the log2 ratio of the observed and the expected normalized counts. High insulation is thus characterized by lower log2(obs/exp) ratio. Note than in Figure 4 the plots are oriented based on the strand of the gene.

In order to calculate the average enrichment of contacts around genomic features (for example ChIP-seq peaks), we first merged adjacent peaks if they were separated by less than 1Kb, and constructed intrachromosomal pairs of intervals. For 5′ to 3′ gene analysis (Figure S5G) matched pairs were used (i.e., belonging to the same gene). We then classified these pairs into IntraTAD pairs between 200Kb and 2Mb, inter-TAD pairs that cross TAD boundaries but are either at close-*cis* range (from 200Kb to 2Mb), or alternatively separated by long distance (2Mb to 10Mb) – “long-range.” We then extracted the observed and the expected contacts in a 40x40Kb window (unless otherwise specified) and processed them as described above. Strong contacts are characterized by a higher log2(obs/exp) ratio. Note that for analysis involving enhancer-promoter interactions, we oriented the resulting plots based on the strand of the gene. In order to maximize resolution, we performed this analysis using the pooled data.

To quantify the total enrichment of contacts over features, or as a function of the genomic distance between the pairs, we used cis-decay curves as described above, but only taking into account Hi-C contacts that fall within a 5Kb bins centered on the genomic features of interest (adjacent peaks located less than this distance were merged). In order to estimate biological variability, we then calculated the log2 ratio of the observed and the expected contacts per replicate either for all intra- (pairs are separated at least 10Kb and not more than 1Mb) or inter-TAD (pairs are between 1Mb and 100Mb) cis-contacts, or as a function of the distance.

#### Average TAD and Intra- or Inter-TAD Contact Enrichment

To calculate the average TAD contact enrichment map, we extend each TAD interval (with a length x) both upstream and downstream by x. We then generated a 100x100 grid from the beginning until the end of the extended 2D interval. As each TAD has a different length the constructed grid had varying bin sizes. We then calculated log ratio of the observed and the expected contacts in each bin of the 100x100 grid per TAD. Figure 3A represents the mean values in each grid bin across all TADs per cell type.

To calculate the log ratio of contacts outside of domains, we constructed two sets of 2D intervals. Upstream of the domain start (start – domain length) until the middle of the domain and downstream of the domain end (end + domain length) until the middle of the domain (see Figure S4A for an illustration). The intervals were adjusted by 10% in order to avoid overlapping with domain boundaries and the total number of the observed and expected contacts per TAD was extracted.

#### FISH

We generated probes using PCR fragments of 1.1 to 1.7 kb amplified from mouse genomic DNA (Table S4). Probes were labeled using the FISH Tag DNA Kit with Alexa Fluor 488, 555, and 647 dyes (Invitrogen life technologies). 3D FISH was adapted from (Bantignies et al., 2011). Briefly, E14Tg2a cells were differentiated as described above and fixed overnight with 4% PFA in PBS at 4C. Cells were treated with PBS, 0.5% Triton for 10 minutes, washed in PBS, incubated with 0.1M HCl for 10 minutes, washed in 2XSSCT (2XSSC, 0.1% Tween20) and incubated for 30 minutes in 50% Formamide, 2XSCCT. 20 to 25 ng of each FISH probes were mixed with 4 μg (for double FISH experiments) or 5 μg (for triple FISH experiments) of Cot-1 DNA (Invitrogen life technologies, Cat No. 18440-016) and 0.8 μL of RNase A (10 mg/mL). This probe mixture was diluted in FISH hybridization buffer (FHB: 50% Formamide, 10% Dextransulfat, 2XSSC, Salmon Sperm DNA 0.5 mg/mL) always keeping at least three times more FHB buffer volume than probe mix volume (for a total volume of approximately 20-25 μL). Probes were initially denatured at 80°C for 10 minutes, cooled in ice, and added to the coverslip before sealing on a slide with rubber cement. Cell DNA and probes were co-denaturated at 80°C for 20 minutes and hybridization was performed at 42°C overnight in a humid dark chamber. Cells were then washed 3 × 5 minutes at 60°C in 2XSCCT, 3 × 5 minutes at 37°C in 2XSCC, 3 × 5 minutes at 45°C in 0.1XSCC, and 2 × 5 minutes in PBS before DNA counterstaining with DAPI (final concentration at 0.1ng/μL in PBS). After final washing in PBS, coverslips were mounted on slides with Vectashield (Clinisciences).

Images were acquired using a Leica SP8-UV microscope (with a 63X/NA 1.4 oil immersion objective), Montpellier Rio Imaging (MRI) facility. Image analysis was performed with Imaris software (Bitplane, Switzerland) and its XT module. After the application of a Gaussian Filter (sigma = 1 pixel), FISH signals were segmented in 3D and their center of mass were calculated. For double FISH experiments, the distances between closest neighbor’s center of mass were calculated (with a maximum distance cut off set at 4μm). For triple FISH experiments, the distances were calculated from one FISH center of mass to the two other FISH closest centers of mass (with a maximum distance cut off set at 2μm for both distances). Only FISH fluorescence signals within DAPI 3D-segmented object were considered for the analysis.

For nuclear mean radius calculation, DAPI channels of images were separated and projected along Z axis using average intensity, before analysis with Cell Profiler software. Objects were segmented using automatic thresholding strategy and the mean nuclear radius of the ES, NPC and CNs population were calculated (ES = 1.73 ± 0.28; NPC = 1.39 ± 0.24; CN = 1.43 ± 0.20). In order to control for the global change in nuclear size in Figures S4H and S4I, we normalized the pairwise FISH distances by multiplying by a cell-type specific factor, defined as the mean nuclei radius in that cell type, divided by the average of all three cell types. We refer to these distances as normalized 3D distance.

#### Identification of Enhancers and Enhancer-Promoter Interactions

To identify enhancers, we used H3K27ac peaks, filtering out regions that were less than ± 5Kb away from known Tss. Peaks were merged if they were located less than 1Kb apart. The ratio between the maximum enrichment of H3K4me1 to H3K4me3 and H3K27ac to H3K4me3 in each putative enhancer region was calculated and only enhancers with H3K4me1/H3K4me3 > 1 and H3K27ac/H3K4me3 > 1 were retained. Enhancers were also further subdivided into CTCF^+^ versus CTCF^-^ based on the presence of a CTCF binding site within ± 5Kb of the enhancer. In order to rule out the contribution of CTCF or promoter-promoter interactions when using average HiC maps, we removed regions, where there was another Tss in the window (±20Kb), filtered out promoters, where there was a CTCF binding site in the window, and oriented the resulting maps based on the direction of transcription.

In order to calculate an Enhancer-Promoter (EP) interaction score, we constructed 5Kb pairs (centered on the respective genomic feature) between enhancers and promoters maximum 2Mb apart in a given cell type. We then extracted the maximum Hi-C interaction score in each cell type within that 5x5Kb window if it was supported by at least 10 contacts (pooled data). For Table S3, we report the Hi-C score between all (Tss-filtered only) pairs of enhancer-promoters, supported by at least 10contacts and with a Hi-C score of > = 30. To calculate the regional enrichment of enhancer-promoter interaction, for each pair we randomly permuted the enhancer anchor 250 times (while keeping the Tss), requiring that the new position is inside the same TAD and that is at least ± 25Kb away from any known Tss or another enhancer.

#### Comparing Enhancer-Promoter Interactions with Gene Expression

To examine EP interaction on developmentally regulated genes, we selected genes that are expressed in at least one condition (FPKM > 1) and have a fold change of at least 4 and clustered them using k-means (k = 8). We then selected only significant EP pairs (Hi-C score > 30) per gene in the cell type where the gene had maximum expression. For example, for the Nanog gene, we considered only the pool of ES enhancers when selecting E-P pairs. We then calculated the average gene expression per cluster (mean ± SEM), the average EP interaction strength per cluster for the EP pairs as defined above (mean Hi-C score ± sem), and the correlation between gene expression and EP interaction strength per gene. As control, we used the shuffled EP pairs as defined above. To determine the gene specificity score we used the method as described (Javierre et al., 2016), but substituted the CHiCAGO score for the E-P Hi-C interaction score as defined above. We calculated the gene specificity score separately based on expression data or HiC interaction score using the expression values or all E-P interaction Hi-C scores to construct the distance matrices.

### Data and Software Availability

#### Software

The R package to compute the expected tracks and the Hi-C scores is freely available at: https://bitbucket.org/tanaylab/shaman. Processing of the raw-reads is done using https://bitbucket.org/tanaylab/schic2 as described above.

#### Data Resources

The accession number for the data reported in this paper is GEO: GSE96107.

## Author Contributions

B.B. and G.C. conceptualized the study. B.B. performed Hi-C, ChIP-seq and RNA-seq, and the *in vivo* experiments and analyzed the data. Q.S. performed and analyzed the FISH. G.L.P. contributed to the Hi-C analysis. L.F. established the CRISPR-dCas9 cell lines. N.M.C., Y.L., and A.T. established the Hi-C analytical framework and contributed to the Hi-C analysis. X.X. and X.L. performed the high-throughput sequencing at BGI. J.P.H. housed the animal strains. B.B. and G.C. wrote the manuscript with input from all authors. For further work, please see http://orcid.org/0000-0002-7502-9399 (B.B.) and http://orcid.org/0000-0003-3709-3469 (G.C.).

## Supplemental Information

Table S1. Summary Statistics for the Dataset Generated in This Study, Related to Figure 1

Table S2. Genomic Coordinates and Compartment Identity of the Topological Domains in Different Cell Types, Related to Figures 1 and 2

Table S3. Hi-C Interaction Score of Detected Enhancer-Promoter Contacts, Related to Figure 7 Tab 1 based on enhancers identified in ES. Tab 2 based on enhancers identified in NPC. Tab 3 based on enhancers identified in CN. Enhancers within 5 kb were merged, and the reported coordinates represent the centers of the intervals

Table S4. List of Primers Used for the FISH, Related to Figures 3 and 5
